# On the Use of Biomineral Oxygen Isotope Data to Identify Human Migrants in the Archaeological Record: Intra-Sample Variation, Statistical Methods and Geographical Considerations

**DOI:** 10.1371/journal.pone.0153850

**Published:** 2016-04-28

**Authors:** Emma Lightfoot, Tamsin C. O’Connell

**Affiliations:** 1 McDonald Institute for Archaeological Research, University of Cambridge, Cambridge, United Kingdom; 2 Department of Archaeology & Anthropology, University of Cambridge, Cambridge, United Kingdom; Museo Nazionale Preistorico Etnografico 'L. Pigorini', ITALY

## Abstract

Oxygen isotope analysis of archaeological skeletal remains is an increasingly popular tool to study past human migrations. It is based on the assumption that human body chemistry preserves the δ^18^O of precipitation in such a way as to be a useful technique for identifying migrants and, potentially, their homelands. In this study, the first such global survey, we draw on published human tooth enamel and bone bioapatite data to explore the validity of using oxygen isotope analyses to identify migrants in the archaeological record. We use human δ^18^O results to show that there are large variations in human oxygen isotope values within a population sample. This may relate to physiological factors influencing the preservation of the primary isotope signal, or due to human activities (such as brewing, boiling, stewing, differential access to water sources and so on) causing variation in ingested water and food isotope values. We compare the number of outliers identified using various statistical methods. We determine that the most appropriate method for identifying migrants is dependent on the data but is likely to be the IQR or median absolute deviation from the median under most archaeological circumstances. Finally, through a spatial assessment of the dataset, we show that the degree of overlap in human isotope values from different locations across Europe is such that identifying individuals’ homelands on the basis of oxygen isotope analysis alone is not possible for the regions analysed to date. Oxygen isotope analysis is a valid method for identifying first-generation migrants from an archaeological site when used appropriately, however it is difficult to identify migrants using statistical methods for a sample size of less than *c*. 25 individuals. In the absence of local previous analyses, each sample should be treated as an individual dataset and statistical techniques can be used to identify migrants, but in most cases pinpointing a specific homeland should not be attempted.

## Introduction

Oxygen isotope analysis is an increasingly popular tool to study past human migrations, and has now been applied to samples from many time periods and locations [1–3]. However, there are a number of points that warrant further investigation and clarification in order to determine the extent to which this technique can be utilised to identify migrants reliably amongst a ‘local’ archaeological sample. In particular: the degree of intra-sample variation that can be expected at a specific geographical location is not well-determined; the best means for identifying statistical outliers (i.e. migrants) in a dataset is uncertain; and the extent to which the spread in human data obscures differences that would be expected between populations from differing geographical locations is unclear. By compiling and analysing published archaeological human oxygen isotope data, this paper considers these three problems.

We assess the typical intra-sample spread by comparing the isotopic variation found within archaeological human samples from the same location. Early studies often assumed that a population consuming local water would have a 2‰ spread due to physiological variation [3, 4], although using this value as a means of identifying migrants is now less common. Nevertheless, the typical spread of biomineral isotope values in a population is an important factor to consider, although difficult to test in a modern population. We examine this assumption using data from the large number of archaeological studies now published.

We consider how best to identify migrants in the absence of sufficient pre-existing comparative data by applying various statistical methods to our dataset and comparing the results. Some researchers have applied statistical methods of outlier identification as if they give a definitive answer. The use of boundaries does provide a clear “cut-off” point, yet the typical distribution of data (including human oxygen isotope values) rarely has clear breaks. Rather, there is usually a continuum of data, and ‘it is often a subjective judgement, as to what constitutes a “sufficient” deviation for a point to be considered an outlier’ [5]. We therefore consider different methods’ strengths and weaknesses with regards to oxygen isotope analysis of human remains.

Finally, we consider how the oxygen isotopic variation in human populations and across geographical regions affects our ability to distinguish between people born in different geographical locations. The maps of oxygen isotope ratios in modern precipitation show that large areas of land have similar values, for example the precipitation oxygen isotope ratios of large parts of Australia, Africa, South America, the Indian subcontinent and southern North America, plus parts of China, South-East Asia, the Near East and southern Europe fall within a few permil of -5‰ [6]. When one further considers that physiological variation and culturally mediated modification of ingested water will increase the variation in human values compared to that of the average local precipitation, plus the possibility of the importation of water and food, we begin to question whether it will be possible to distinguish between locals and migrants. By considering the collated published data by location, we assess the extent to which it is possible to distinguish between individuals from different locations within Europe.

The main concern of this paper—the need to be rigorous in the identification of outliers—is illustrated with regard to human oxygen isotopic values in archaeology, but is equally pertinent to other isotope systems used in geolocation (e.g. strontium, hydrogen, as well as oxygen) and in many fields beyond archaeology, including forensics (human and wildlife) and ecology (e.g. migration patterns).

## Background

### Oxygen isotopes from precipitation to the body

Oxygen isotopes in mammalian tissues reflect the isotope values of the water ingested (as water or from food) at the time of tissue formation [7]. Usually, ingested water closely approximates the isotopic composition of the local meteoric precipitation, which varies geographically and temporally. The reasons for the spatial and temporal variations in the oxygen isotope ratios of precipitation have been discussed in detail elsewhere [8], hence only a brief summary will be presented here. The isotopic variation in precipitation on Earth is largely explained by Rayleigh fraction that occurs within air masses. Water containing ^18^O preferentially condenses out, leaving the vapour phase relatively enriched in the lighter isotopologue. As more ‘heavy’ water is removed from the system, the remaining vapour becomes progressively lighter. The isotopic composition of precipitation at a given location is related to a number of environmental parameters, such as latitude, altitude, distance to coast, amount of precipitation, surface air temperature and season [9, 10]. Of these, two factors are particularly noteworthy—the oxygen isotope value of precipitation is strongly positively correlated with surface temperature at high latitudes and in tropical regions it is negatively correlated with the amount of rainfall [11]. These factors also cause temporal variations associated with past climatic changes in a given location, allowing oxygen isotopes to be used as a palaeoclimatic indicator but limiting the comparability of results for migration studies (e.g. [12, 13]).

The δ^18^O of groundwater generally reflects the average isotopic composition of the precipitation that fell recently in the local source area [14]. Nevertheless, variations between precipitation and groundwater δ^18^O values can be caused by various factors, including: evaporation from surface water; fractionation during water movement through the soil or aquifer; exchange within geologic formations; recharge that occurred in past periods when the isotopic composition of precipitation was different from at present; and recharge from rivers containing water derived from high altitude precipitation [14, 15]. There is also short-term oxygen isotope variation in precipitation—within rain events, seasonally, inter-annually, and decadally [10].

Drinking water is mainly derived from the local groundwater and thus approximates the local precipitation, however ingested water also includes water from food. The exact proportion of water obtained via these two routes is variable, is species-specific and depends upon the amount of water in the foods consumed [16]. Both water drunk and water in food (where it is locally sourced) derive from local precipitation and thus reflect the local climate [17, 18]. However, a number of factors may introduce oxygen isotopic variability into food and water sources. Culturally mediated behaviours can alter the isotope ratio of the water drunk compared to that of the precipitation and groundwater through, for example, boiling, stewing, brewing and vinification [19]. Milk consumption is also known to have an effect on consumer δ^18^O, both human (the “breastfeeding effect” [20]) and animal milks, as milk is enriched in ^18^O relative to local water [21], due to the producer’s higher body temperature. Imported food and drink may have a different δ^18^O value compared to locally-sourced ingested water [16, 22].

The δ^18^O of body water is controlled by the δ^18^O of water entering and leaving the body. Variations in these inputs and outputs control the fractionation between environmental water and body water [17, 23, 24]. A direct quantitative relationship between the oxygen isotope composition of bone phosphate and environmental water has been shown for many mammals, including humans [17, 18, 25, 26]. The δ^18^O of mammalian carbonate and phosphate are linked to that of body water at constant body temperature, although the relationship is not fully characterised [7] and may be variable, as oxygen isotopic fractionation in humans can be moderated by a number of physiological factors, such as activity, smoking, metabolic rate and disease [27–31].

Oxygen isotopes can be measured in any human or animal body tissue [12, 18, 32–34], but in archaeological contexts the measured tissues are most often biological apatites (bioapatite) in bone or tooth enamel. In bioapatite, oxygen isotopes can be measured in carbonate or phosphate; while phosphate is less susceptible to diagenetic alteration, carbonate analysis is simpler, quicker and cheaper (and also allows the determination of δ^13^C values, reflective of diet). As tooth enamel is relatively resistant to diagenetic alteration, carbonate analysis of tooth enamel bioapatite is generally considered valid, while carbonate analysis on bone should be avoided as it is subject to diagenetic alteration [35–38].

### Oxygen isotopes as a method of identifying migrants

#### Assumptions of the Method

Oxygen isotope analysis has been employed since the early 1990s as a means of identifying migrants in archaeological samples [39]. As there are geographical variations in the isotope ratios of environmental water, we can identify individuals whose oxygen isotope ratios are different from those expected at the place of burial. Thus the technique is based on two assumptions: 1. that precipitation in different geographical areas have different oxygen isotope ratios; and 2. that these signals are incorporated within human bodies in such a way that the differences in oxygen isotope ratios of precipitation between areas are preserved. The former of these assumptions is well studied (for example, by the Global Network of Isotopes in Precipitation project [40]) with mean annual oxygen isotope distribution maps showing clear variation across the globe, although there are large swathes of land that have similar oxygen isotope ratios in their precipitation [6]. These have been used as reference datasets for archaeological applications of oxygen isotope values as a marker of human movement.

Less well studied is the second assumption, that the local precipitation oxygen isotope signals are incorporated within human body tissues in such a way that differences in oxygen isotope signals between geographical areas are preserved. This requires that the oxygen isotope values of drinking water mirror that of the average local precipitation and that the incorporation into human body tissues is similarly faithful, such that variation introduced through human physiology does not obscure geographical patterns. There are various reasons why the former may not hold true in all cases, including that groundwater has a different oxygen isotope ratio to precipitation, the consumption of non-local food and drink, and human alteration of water oxygen isotope values through brewing and so forth, as mentioned above. Furthermore, the seasonal and inter-annual oxygen isotope variation in precipitation will be reflected (though likely dampened) in drinking water and food sources, which will introduce variation into consumer tissues relating to the particular period in which the tissue formed (and remodelled). Such signals are clearly seen in faunal hypsodont teeth [41, 42], and it is probable that some such temporal isotopic variation occurs in human teeth, which mineralise relatively rapidly in an incremental fashion. Even presuming that a population is consuming food and water that is isotopically indistinguishable from that of the local precipitation, there is likely to be some degree of physiological variation introduced (i.e. ‘noise’ added to the dataset) through intra-individual variability in body processes, as well as potential isotopic differences between different bioapatite matrices (tooth *vs*. bone), as has been observed for different protein pools within the body [43]. Beyond this, the method of sampling when taking a bulk enamel signal to gain an ‘average’ δ^18^O value may well influence the measured isotopic result [44].

Because of these issues, and the difficulties associated with conducting modern studies on bioapatite, it remains unclear what spread of oxygen isotope values is to be expected within an archaeological sample. It is therefore difficult to identify the individuals who do not have typical δ^18^O values for a given location, that is, the migrants: in mathematical terms, it is difficult to distinguish outlying values (anomalies) from the noise in the dataset. It is also not possible to assess the validity of identifying ‘homelands’–while the oxygen isotope precipitation maps give a clear indication of modern annual average precipitation δ^18^O values, this picture will be ‘blurred’ to a greater or lesser extent by the variation inherent in a human population. It is these three problems—the variation in a sample of a population, how to identify outlying values, and the amount of variation in human values due to geography—that we address in this paper.

#### Methods used to Identify Migrants: 1. Prior Knowledge of Variation

There are several approaches by which bioarchaeologists identify someone as a migrant using bioapatite oxygen isotope data. These fall into two main groups—those that require prior knowledge about oxygen isotope values at geographical locations (such as that of the burial location, as well as possible originating locales) and those that are based on statistical methods of identifying individuals who are outliers within a sample.

The first set of approaches assesses whether an individual’s oxygen isotope value matches that expected for someone born and raised in the local area. This has been typically done in two ways: firstly, assessing the degree to which the estimated water oxygen isotope value that an individual consumed in childhood is concomitant with that of the local water oxygen isotope value (e.g. [45]), and secondly, the degree to which the individual’s oxygen isotope value matches that of previously analysed individuals born and raised in the local area (e.g. [46]). Both of these approaches require knowledge of the oxygen isotopic ‘local range’ or ‘local signal’, either in water or in human bioapatite, and are often used in conjunction with each other.

Where a comparison is made to the local water oxygen isotope signal, it is good practice that the definition of this ‘local signal’ considers all available water, including any minor water sources that may have been present in the past, as well as water that has been treated in such a way that the oxygen isotope value may have changed (for example boiled or used to make alcoholic beverages [19]). The ‘local water signal’ is usually determined from estimations based on modern water (such as the GNIP database [40], see also [47]), with the limitation that this does not account for climatic changes altering the oxygen isotope values of the precipitation through time. A ‘local signal’ may also be derived from local faunal δ^18^O values, converted to a water δ^18^O equivalent [48], although this method introduces uncertainty associated with conversion of data and assumes that animals and humans have similar access to the same water source(s). For comparisons with a ‘local signal’, there needs to be a good knowledge of the relationship between the water isotope values and the body tissue isotope values, ensuring that it is relevant to the area and isotope values in question, such that the latter can be converted to the former. There are several published conversion equations for humans [16–18, 35, 45, 49, 50], which lead to differing δ^18^O_water_ values, making interpretation of the results problematic. There are also significant concerns with error propagation in conversions of tooth enamel oxygen isotope values to those of water [51]. These problems have been debated elsewhere and the reader is referred to [50, 51] for further discussion.

A related approach is to use previously published human δ^18^O values to define a ‘local range’ against which to compare the target human oxygen isotope values [46]. This is a more robust method, since it avoids the problems inherent in the conversion of human oxygen isotope values, but can obviously only be used in regions where a significant number of studies have previously been published, such as the UK. If such comparative data are unavailable, as is the case for large regions of the world (see below), then this method is not applicable.

Implicit in many analyses using a ‘local range’ is an assumption about the expected spread of isotope values in a human population drinking water from the same source. A spread of 2‰ in tooth enamel phosphate δ^18^O was suggested from early studies [3, 4], however it is now timely to review this figure using the greater number of publications that are now available.

#### Methods used to Identify Migrants: 2. Statistical methods of identifying outliers

The second set of approaches is statistical, that of identifying outliers in a measured sample of a population, such that we can say that their tooth enamel oxygen isotope values do not appear to come from the same “distribution” as the others of the burial sample. The basic premise behind these techniques is that for a univariate numeric dataset, such as a set of human bioapatite oxygen isotope values, if we can describe the underlying population distribution that represents the majority of the data, then we can spot those observations that don’t fit, and identify them as outliers. Mathematical definitions of outliers include “an observation which deviates so much from the other observations as to arouse suspicions that it was generated by a different mechanism” [52] or “one that appears to deviate markedly from other members of the sample in which it occurs” [53] or “unusual observations that do not seem to belong to the pattern of variability produced by the other observations” [54].

The first step then is to describe the underlying population. However, we must recognise that when we are analysing human oxygen isotope values from an archaeological site we have the numeric data of *both* (what we assume is) the underlying population *and* any outliers, often in relatively small numbers. It is therefore imperative that we describe the population in a way that is not influenced by the outliers, but without the knowledge of which data points are outliers, and that this description is applicable to relatively small sample sizes.

We can describe a sample using “measures of scale”–statistical estimators that allow us to assess the dispersion of a set of numeric data. Conventional measures of scale, such as variance (Var) and standard deviation (SD) are very sensitive to outliers. Robust measures of scale (please see S1 Appendix for definitions), on the other hand, such as the interquartile range (IQR) and the median absolute deviation from the median (MAD) are relatively insensitive to outliers. Thus, to describe a dataset that can include both the underlying population and any potential outliers (in the case of human bioapatite oxygen isotope data), we should use robust measures of scale (IQR and MAD), not conventional ones (Var and SD).

Once the underlying population has been described, there are a wide variety of outlier identification methods available [5]. Of these, the classical probabilistic and statistical techniques of outlier labelling are the most simple for univariate data, and have been widely applied in archaeological applications of oxygen isotope data. Such methods are based on determining boundaries calculated from the same statistic by which we describe the sample, and then using these boundaries to identify outliers (e.g. standard deviations [55] or the IQR [56]). These methods are powerful when used in appropriate circumstances, however it is critical to note that the number of outliers identified will change according to the method used, the sample size and the distribution type of the data [57].

Outlier labelling methods that use conventional measures of scale, such as two standard deviations from the mean, are sensitive to extreme values, whilst those that use robust measures are much less sensitive, such as the IQR (Tukey’s boxplot method) and the median absolute deviation from the median (MAD [58]). The implications of this sensitivity (or not) to outliers are on the prevalence of false positive and false negative identifications. Labelling methods that rely on measures of scale that are sensitive to outliers are more likely to give false positive identifications (thus overestimating outlier percentages) whereas those that are insensitive or robust to outlier values are more likely to give false negative identifications (thus underestimating outlier percentages). When using such statistical methods of outlier labelling, researchers must recognise that there will always be a trade-off between sensitivity to outliers and false positive or negative identifications.

Classical probabilistic and statistical techniques of outlier labelling are powerful with large datasets, but there can be difficulties when applying them to small sample sizes. Sample size is often overlooked as a factor in describing underlying population distributions, yet can have a significant effect on inferences drawn [59]. For a given method, a greater proportion of outliers are found with small sample sizes than with large ones [57].

It is critical to realise that the choice of an appropriate statistical method also depends on careful assessment of the assumptions inherent in each method, as well as of the data to be analysed. All methods assume a single unimodal underlying population (i.e. no bimodality, such as might occur from consumption of two or more water sources that are isotopically different). Use of a labelling method based on standard deviations assumes that the data are parametric, and in larger sample sizes will tend towards identifying 5% as outliers. The IQR and the MAD methods can be used on non-parametric data, but considers the sample as coming from a single population, with little consideration of skewness or potential bimodality in the population.

Clearly all of these methods have their strengths and weaknesses, but it remains unclear which, if any, of these statistical methods is the most appropriate for identifying migrants in the archaeological record using oxygen isotope analysis. Here, we test the different methods on the published datasets on human oxygen isotope values.

#### The Identification of Potential Homelands

Most studies have not used oxygen isotope data to identify homelands in isolation, but rather rely on other archaeological or historical information to limit the possibilities. The isotopic methodology is simple—matching the isotope value obtained for the migrant in question to known geographical patterning in water oxygen isotope values (whether from large-scale studies such as GNIP, or by undertaking water measurements as part of the study) or sometimes through comparison to previous isotope studies of human biomineral. Both of these approaches have limitations, the former in the difficulties of comparing data from human biomineral to that of water, and the latter in requiring extant comparative data. In both cases the bioarchaeologist can combine prior assumptions based on archaeological evidence with the scientific data to limit the geographical possibilities in some way. While in most cases these prior assumptions are valid, the interpretations drawn can only be described as the most parsimonious explanation of the isotope data, as the values themselves could be interpreted in other, admittedly less plausible, ways given the large degree of overlap in (modern) oxygen isotope values in precipitation globally [6]. Here, we undertake a geographical comparison of human oxygen isotope values in order to quantify the spatial patterning in human oxygen isotope values.

### Methodology

We compiled human biomineral oxygen isotope data from published papers and book sections, over a period beginning in mid-2009 and finishing on 5^th^ June 2015. We included studies reporting data from specimens of tooth enamel carbonate, tooth enamel phosphate, bone carbonate and bone phosphate taken from humans (modern and archaeological) dating from the Holocene. We excluded (a) studies where the measured value was not reported (only a ‘converted’ value); (b) analyses from human hair and other proteinaceous material (since initial assessments suggested that they were not comparable to those from bone and teeth apatite); and (c) individuals dating from before the Holocene. The database used to find the information for this study was primarily Web of Knowledge, combined with more limited searches on GoogleScholar. Various combinations of the following search terms were used: oxygen, isotop*, human, migration and mobility. The refine function on Web of Knowledge was used to limit the Research Areas to Archaeology and Anthropology. It should be noted that this study should not be considered exhaustive, but rather is biased towards data published in English, searchable on Web of Knowledge.

The isotope values were entered into an Excel spreadsheet along with published ancillary information about the site and skeleton (including location data and material type). The material analysed (i.e. tooth/bone) was recorded and those specimens from teeth were categorised into teeth unlikely to be affected by any isotope signal from breastfeeding (permanent premolars, 2^nd^ and 3^rd^ molars) and those more likely to be affected (incisors, canines, 1^st^ molars and deciduous dentition). Where an individual was represented by more than one specimen, only one δ^18^O measurement was entered into the spreadsheet (normally the tooth least likely to be affected by breastfeeding, i.e. one of the permanent premolars, or 2^nd^ or 3^rd^ molars). We use the term ‘sample’ to describe the isotopic dataset from a collection of specimens taken from a single site, and define ‘site’ in such a way that specimens taken from the same modern settlement are grouped as one site in order to increase the sample size per site: for example all of the data from various sites in York [60–62] are considered to be one site.

All isotope results were recorded in the spreadsheet in their measured form, that is before any conversions were performed in the publications from which the data were collected. We then converted the data, where necessary, using the following equations, from carbonate oxygen VPDB to carbonate oxygen SMOW:

$$\delta^{18}O_{\left(VSMOW\right)}=\;1.03091\;\times \;\delta^{18}O_{\left(VPDB\right)}\;+30.91$$ (1)

[63]:

From carbonate to phosphate:

$$\delta^{18}O_{PO4}=\;1.0322\;\times \;\delta^{18}O_{CO3}\;-9.6849$$ (2)

[35]:

The use of two preliminary conversions does introduce some degree of uncertainty, but we do not formally consider any error propagation. Whilst these two conversions each have statistical errors associated with them, to do with defining the line of best fit through the data points [51], their correlation coefficient r^2^ is very close to 1, meaning the associated errors are minimal. Similarly, measurement errors on oxygen isotope values are typically negligible compared to the calibration errors.

All the data in this paper are compared as phosphate (or phosphate equivalent) oxygen isotope values (δ^18^O_PO4_) on the delta scale relative to the international standard VSMOW in units of permil (‰). We do not convert them to drinking water equivalents due to concerns with error propagation [50, 51], as well as with the choice of the most appropriate published equation to convert human phosphate δ^18^O to δ^18^O_water_ [16–18, 35, 45, 49, 50].

To consider typical intra-sample variation in δ^18^O_PO4_, we calculated multiple measures of scale. For each site, the range, standard deviation (SD), inter-quartile range (IQR) and median absolute deviation from the median (MAD) were calculated and then considered as a function of the number of analysed individuals (N). For MAD, we used two variants, where the MAD was scaled assuming normality of the data (MAD_norm_), and where it was scaled using a value of b set to 1/Q3 (MAD_Q3_) which does not assume normality of the data (see S1 Appendix for full details).

We used three statistical techniques to identify outliers within the analysed sample from each site. We defined boundaries based on three measures of scale for each sample: ±2 standard deviations from the mean, Tukey’s IQR method (1.5×IQR beyond Q1 or Q3), and 3 times the median absolute deviation from the median (±3×MAD from median, using both variants). We refer to these methods as 2SD, 1.5IQR, 3MAD_norm_ and 3MAD_Q3_ in the following text. We also contrasted this with the 2‰ spread method (outliers are values >1‰ from the sample mean), thus making a total of five methods of outlier identification for each individual within each sample. Outlier identifications were only carried out on sites with sample sizes greater than four, as sample sizes less than this can give dubious statistical results and because MAD cannot be calculated for less than five data points.

The European data were compared by modern country (despite the limitations of this spatial approach to archaeological data), latitude, longitude, altitude and modern (interpolated) precipitation oxygen isotopic values. Both coordinates and altitudes were taken from www.geonames.org or GoogleEarth. For comparisons, the sites were grouped (binned) either into 5° grid squares (for latitude and longitude) or 150masl intervals (for altitude) for analysis. Where possible, the interpolated modern site-specific precipitation δ^18^O values (hereafter δ^18^O_MAP_) were taken from [64, 65] and extracted using ArcGIS, otherwise they were taken from www.waterisotopes.org [6, 64, 65]. For comparisons, the human isotope data were grouped (binned) by the δ^18^O_MAP_ values in 1‰ intervals (i.e. all the human values that fall into areas with precipitation values between -10 and -9‰ constitute one group).

Statistical comparisons were performed using IBM SPSS Statistics version 22 for Macintosh and RStudio Version 0.99.473 for Macintosh. Samples were tested for normality using histograms and Kolmogorov-Smirnoff and Shapiro-Wilk tests, and for equality of variance using Levene’s tests. For all comparisons, we used non-parametric tests, as in each case some of the data violated the assumptions of parametric data. Correlations were calculated using Spearman’s Rho tests, which has the test statistic r_s_. When comparing between groups based on geographical considerations, we used the rank-based Kruskal Wallis test, with the test statistic H. The data were also plotted geographically using ArcGIS version 10.2.2 for PC.

## Results

Here we first assess the variability of the dataset, then various means for identifying outliers within it and finally consider geographical patterning.

4085 analysed individuals from 316 sites are included in the dataset. Spatially, they range from -36.77° to 69.22° latitude and -166.73° to 168.37° longitude. In date they range from *c*. 5000BC to modern, and are from 57 modern countries. The material types found are bone carbonate (626 individuals), bone phosphate (276 individuals), tooth enamel carbonate (1862 individuals) and tooth enamel phosphate (1321 individuals). Full information about each specimen is recorded in the full dataset which has been deposited in the University of Cambridge data repository (https://www.repository.cam.ac.uk/handle/1810/252773).

The results, analyses and discussions presented below are limited to a subset of the full dataset (analyses of 2012 individuals), except where specifically stated. This subset (hereafter referred to as ‘Post-Infant Dentition’ subset or ‘PID’) includes only analyses of teeth unlikely to have been affected by breastfeeding (permanent premolars, 2^nd^ and 3^rd^ molars). The PID subset does not include any data from bone or any data from teeth that may have formed during breast-feeding (incisors, canines, 1^st^ molars and deciduous dentition), thus reducing the two problems of tissue comparability, and confusion between local food/water signals and life-history traits (infant feeding practices). For the PID data-subset, statistical methods of outlier identification were applied only to 1873 individuals from 88 sites where N>4. The equivalent tables and figures for the same analyses on the entire dataset (i.e. all teeth and bone, from 4085 individuals) are presented in the supplementary information (S1 to S9 Figs). In all our analyses we combined phosphate and carbonate data from teeth. Although phosphate is considered to be less susceptible to diagenesis and therefore an analytically preferable material to carbonate, the use of only phosphate analyses would have significantly restricted the sample size, given that there is a greater corpus of carbonate data than of phosphate. Where absolute values are mentioned in the text, these are phosphate or phosphate equivalent data, as discussed above. No attempt has been made to account for temporal variation (as per [13]), as this would limit the sample sizes available for comparison. We do not expect this to have a significant impact on our results as all sites date to the Holocene, however we would raise this as an avenue for future research when the available sample size has increased. For the majority of our statistical analyses, we examine intra-site variability, therefore the question of inter-laboratory comparability does not arise, although we acknowledge that such factors as sample preparation and machine calibration may well be significant [66–68]. Where we do carry out analyses of the dataset as absolute and not relative values, the data are examined at a large scale, so we consider that minor inter-laboratory differences are unlikely to have a significant effect.

### Degree of Intra-Sample Isotopic Variability

**Fig 1:**
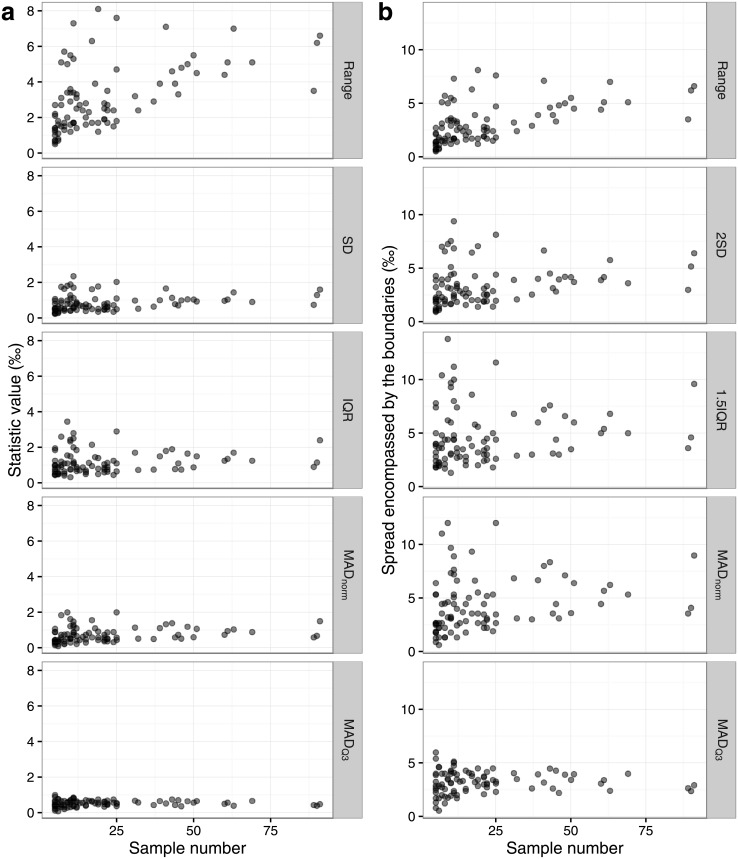
Comparison of the different measures of scale for each site in the Post-Infant Dentition data-subset plotted by site sample size, for all sites where N>4. On the left, (a) the statistic value for the range, SD, IQR and MAD_norm_ and MAD_Q3_. On the right, (b) the spread encompassed by the range, and the boundary values of the methods of 2SD, 1.5IQR, 3MAD_norm_ and 3MAD_Q3_. Criteria of each method (2SD, 1.5IQR, 3MAD_norm_, 3MAD_Q3_) as defined in the text.

All measures of scale calculated for each site vary considerably, as do the spreads encompassed by the calculated boundaries of the different outlier identification methods (i.e. 4SD for the 2SD method, 4IQR for the 1.5IQR method and 6MAD for both the 3MAD_norm_ and 3MAD_Q3_ methods) (Fig 1, S1 and S2 Tables). Intra-site range is very varied, up to a maximum of 8.1‰ (at Pica 8, Peru, N = 19 [69]). Only 33 out of 88 sites have a range of less than 2‰ overall and all of those have a sample size of 25 or less. Across all sites, SD, IQR and MAD_norm_ are all more consistent than range but all three statistics show a lot of scatter below sample sizes of 25. The MAD_Q3_ is never greater than 1‰, and is the most consistent across sample sizes.

Range is strongly positively correlated with sample size (r_s_ = 0.601, P<0.001, N = 88). SD (r_s_ = 0.351, P = 0.001, N = 88), IQR (r_s_ = 0.222, P = 0.038, N = 88) and MAD_norm_ (r_s_ = 0.377, P<0.001, N = 88) are weakly positively correlated with sample size. MAD_Q3_ is not correlated with sample size (r_s_ = 0.111, P = 0.303, N = 88).

**Fig 2:**
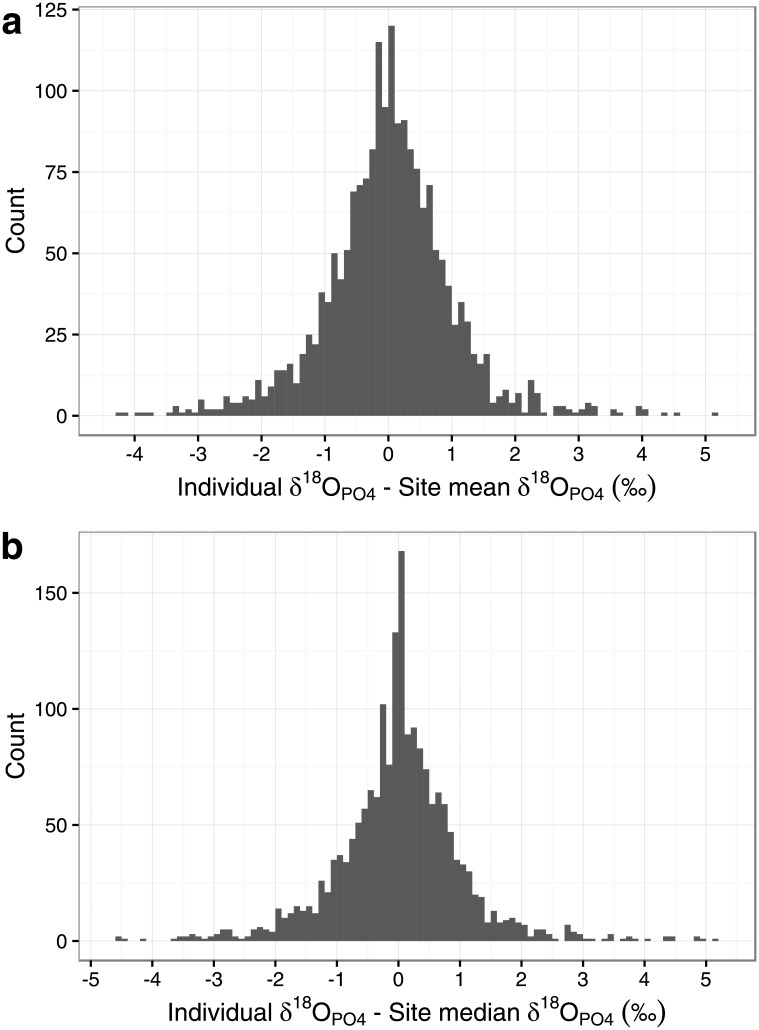
Histogram of the difference between each individual’s oxygen isotope value and (a) the site mean oxygen isotopic value or (b) the site median oxygen isotopic value, for all specimens from sites where N>4 in the Post-Infant Dentition data-subset.

To assess the degree of intra-sample variability for all sites together, we considered the difference between each individual data point and its sample (site) mean and median, for data from sites where N>4 (Fig 2). In each case, the data form a symmetrical leptokurtic distribution, i.e. with tails which are slightly fatter than found in a normal distribution, approximating more closely a Laplace distribution (scale parameter σ = 0.7‰ for the difference from site mean, 1.04‰ for the difference from site median). The overall range is greater than 9‰ for both distributions, with 1415 of 1873 (75.5%) data points within 1‰ of the site mean and 1433 of 1873 (76.5%) data points within 1‰ of the site median.

### Comparison of Statistical Methods of Identifying Outliers

**Fig 3:**
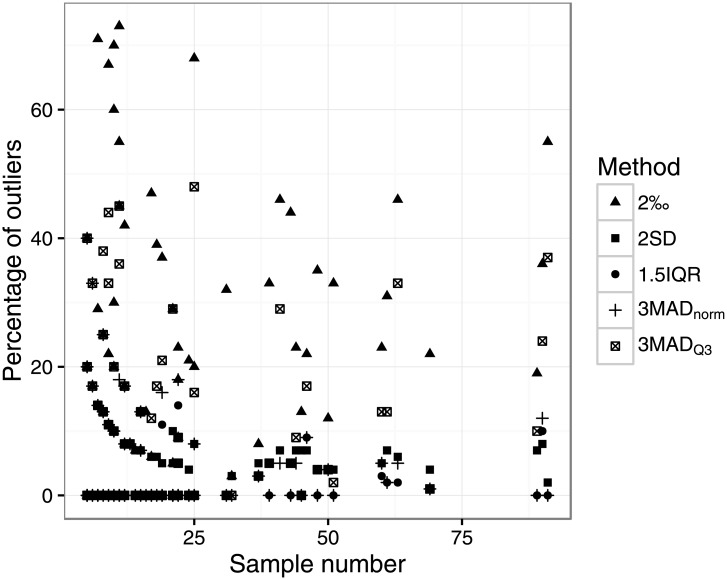
The proportion of outliers found at each site using the five different outlier identification methods, plotted by site sample size, for all sites where N>4 in the Post-Infant Dentition data-subset. For some of methods, there are multiple apparent curves for sample sizes between 5 and 25. This is an artefact of producing estimates of either one, two or three outliers within the sample, producing “curves” of 1/x where x is the number of outliers (i.e. 1, 2 or 3).

From Fig 3, it is clear that the percentage of outliers identified at each site varies considerably depending on the method used. Sample size plays a significant role, especially at smaller sample numbers (less than 15 or even 25).

The use of a 2‰ spread around the sample mean was more likely to identify outliers at a site than the other methods (Table 1). Compared to the other methods, it usually produced the highest percentage of outliers for each sample (Fig 3 and S3 Table) and did so for every site where N≥25, and for 28 out of 32 sites for 11≤N≤24). These outlier percentage estimates were also often very high: 24 out of 88 sites where N>4 (and 11 out of 22 sites where N≥25) had outlier estimates of ≥30%. As noted above, only *c*. 76% of the individuals in the PID database fall within 1‰ of their site mean or median (Fig 2).

**Table 1:** Effect of outlier identification method on the numbers of sites where outliers are identified, for the Post-Infant Dentition data-subset.

Method	Number of sites where outliers identified (of 88 sites with N>4)	% of sites where outliers identified (of 88 sites with N>4)	Number of sites where outliers identified (of 22 sites with N≥25)	% of sites where outliers identified (of 22 sites with N≥25)
2‰	61	69.3	21	95.5
2SD	47	53.4	19	86.4
1.5IQR	26	29.5	12	54.5
3MAD_norm_	33	37.5	11	50.0
3MAD_Q3_	50	56.8	18	81.8

The 1.5IQR and 3MAD_norm_ methods were the methods least likely to identify outliers at a site (Table 1). The 1.5IQR method estimated a maximum of 14% outliers at any one site (max of 10% for N≥25). Considering all sites, the 3MAD_norm_ method identified more outliers per site, especially at smaller sample sizes (Fig 3 and S3 Table); at higher sample numbers (N≥25), the 3MAD_norm_ method identified a similar proportion of outliers as the 1.5IQR method, a maximum of 12%. The two methods are in frequent agreement: they identify the same outliers for 64 of 88 sites (16 of 22 sites where N≥25), and for sites where they do differ, there are never more than two individuals with divergent assessments. Overall, they are in disagreement for only 31 out of 1873 (1.7%) individuals in the PID database.

In contrast, the 3MAD_Q3_ method is more likely to identify outliers at a site (Table 1), and identifies far more outliers per site than either the 1.5IQR or 3MAD_norm_ methods (Fig 3 and S3 Table). These outlier percentage estimates were often very high: 10 out of 88 sites (and 5 out of 22 sites where N≥25) had outlier estimates of ≥30%. It is also rarely in agreement with either 1.5IQR or 3MAD_norm_ estimates: of the 88 sites, the 3MAD_Q3_ estimate differs from those of 1.5IQR or 3MAD_norm_ for 42 and 38 sites respectively (16 and 15 of 22 sites, respectively, where N≥25).

**Fig 4:**
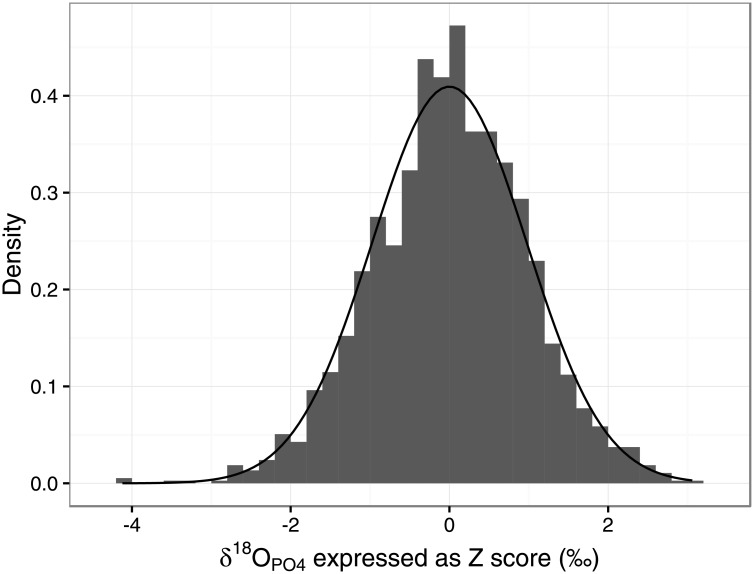
Density plot of each individual’s oxygen isotope value expressed as a z-score for all specimens from sites where N>4 in the Post-Infant Dentition data-subset, with a fitted normal distribution curve overlaid. Z-scores as defined in the text.

The 2SD method produced estimates of outlier percentages from 0 to 13% (between 0 to 8% for N≥25), which is a lower maximum figure than for 1.5IQR, 3MAD_norm_ and 3MAD_Q3_ (S3 Table). However, it identified outliers at many more sites than 1.5IQR and 3MAD_norm_, at similar levels to 3MAD_Q3_ (Table 1). For 19 of the 22 sites where N≥25, there was always at least one outlier identified by this method, even where the overall site range was small (e.g. one outlier at Radovesice, Czech Republic [70], where the overall range was 2.4‰, and two outliers at Khok Phanom Di, Thailand [71], where the overall range was 2.9‰). The use of the 2SD method tends towards the identification of 4.6% of the sample as outliers, since by definition 95.4% of a normally distributed sample around the mean lies within two standard deviations. To assess this for the PID dataset as a whole, we converted individuals’ oxygen isotope values to site-specific z-scores for all sites with N>4: that is, all data points from a sample were normalised to a distribution with a mean of 0 and a SD of 1, such that a specimen with a z-score of 1 has a δ^18^O which is 1 standard deviation above the sample mean. For the 1873 individuals from 88 sites where N>4, a plot of these z-scores shows that overall, the PID dataset z-scores approximate a normal distribution and indeed 86 individuals or 4.59% of the data fall more than 2SD from their site mean (Fig 4).

### Spatial Assessment of Isotope Results within Europe

**Fig 5:**
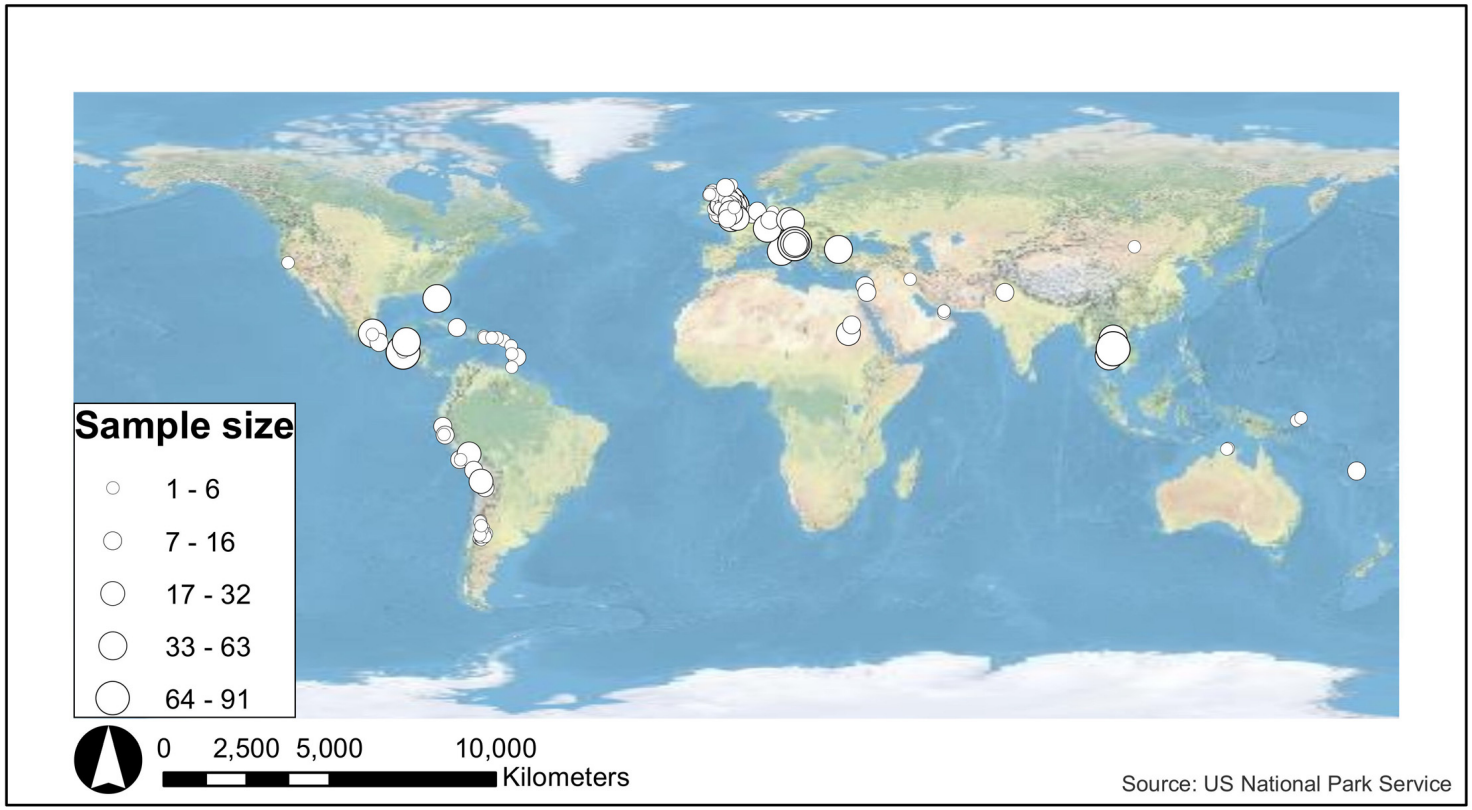
Map of all sites represented in the Post-Infant Dentition data-subset. Map created using ArcGIS version 10.2.2 for PC. Source: US National Parks Service.

**Fig 6:**
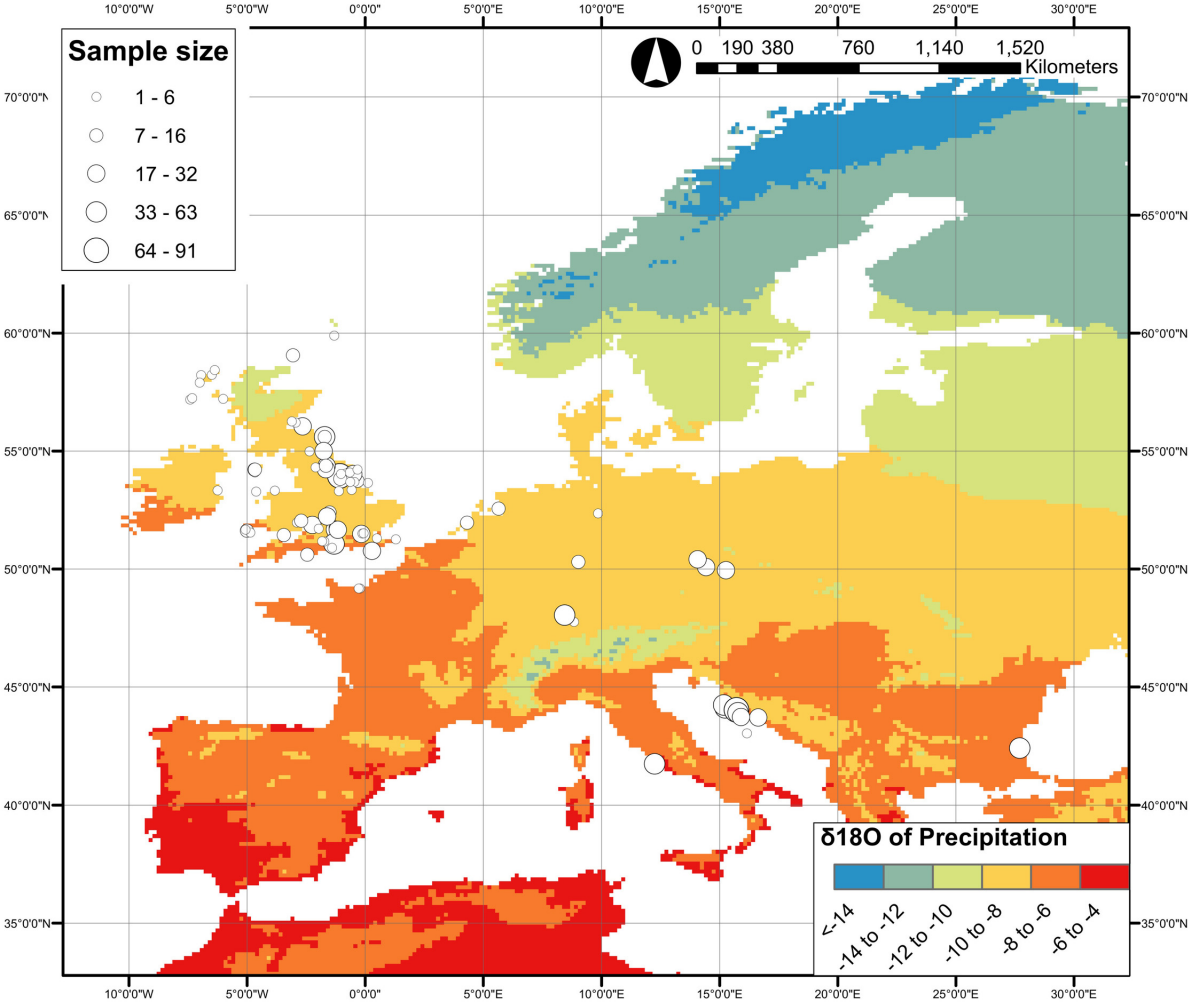
Map of all European sites represented in the Post-Infant Dentition data-subset, with regional modern precipitation oxygen isotopic values indicated. Map created using ArcGIS version 10.2.2 for PC. Precipitation data taken from Bowen 2015 [64] and Bowen and Revenaugh 2003 [65].

**Fig 7:**
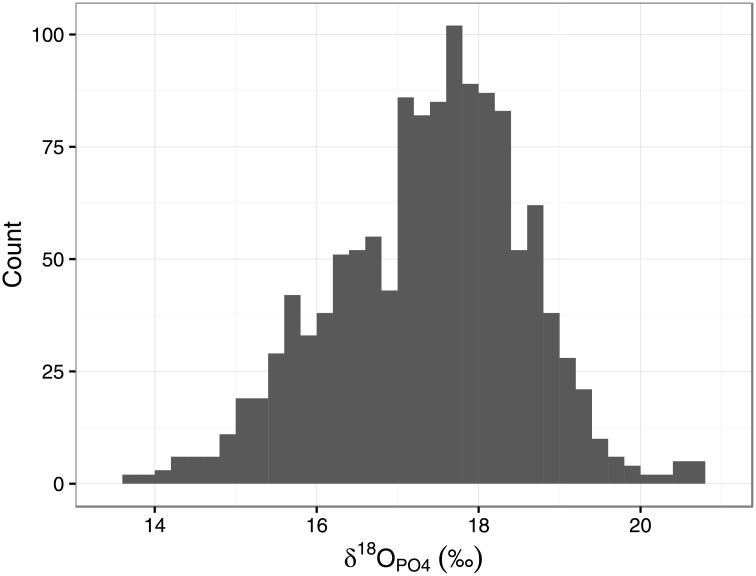
Histogram of oxygen isotope values for all European individuals in the Post-Infant Dentition data-subset.

The sites included in this analysis show clear and expected geographic biases in the dataset with more than half of the PID subset coming from Europe (1266 out of 2012 individuals) (Fig 5). Given the high concentration of data from Europe, and the relatively poor coverage for the rest of the world (including the Americas), we therefore constrain our spatial assessment here to the European data. It should be noted, however, that there are biases within Europe, with the data generally along a north-west to south-east distribution from the UK to Croatia, and the highest concentration in the British Isles (Fig 6). All PID European tooth enamel δ^18^O_PO4_ fall within 13.7 to 20.7‰ (a total of 1266 individuals from 91 sites, S1 Table, Fig 7).

**Fig 8:**
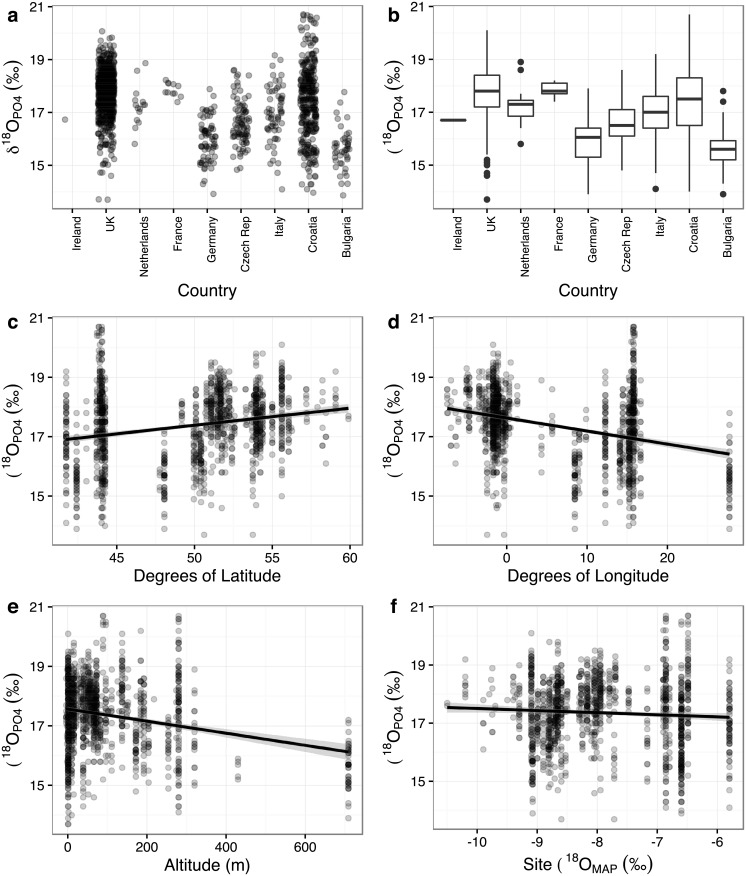
Oxygen isotope values for all individuals from European sites in the Post-Infant Dentition data-subset, plotted by: (a) and (b) country, (c) latitude, (d) longitude, (e) altitude, (f) modern precipitation oxygen isotopic values (δ^18^O_MAP_). (c) Latitude is positively correlated with δ^18^O_PO4_: r_s_ = 0.246, P<0.001, N = 1266. (d) Longitude is negatively correlated with δ^18^O_PO4_: r_s_ = -0.272, P<0.001, N = 1266. (e) Altitude is weakly negatively correlated with δ^18^O_PO4_: r_s_ = -0.092, P<0.005, N = 1266. (f) δ^18^O_MAP_ is not correlated with δ^18^O_PO4_: r_s_ = 0.019, P = 0.494, N = 1266.

We examined the data from Europe by the site-specific parameters of country, latitude, longitude, altitude and δ^18^O_MAP_ (Fig 8). There are statistical differences between the countries’ average values. Although there are statistically significant correlations between δ^18^O and each of latitude, longitude, altitude and δ^18^O_MAP_, these are very weak, with a high degree of scatter in the data. Tooth enamel δ^18^O_PO4_ values between *c*. 16 and 18‰ are found in all of the analysed countries, and across the entire range of latitude, longitude, altitude and δ^18^O_MAP_. Considering the range of δ^18^O_MAP_, 63 of the 92 analysed European sites fall in areas with δ^18^O_MAP_ between -9 and -7‰, and all of the sites have δ^18^O_MAP_ of between -10.5 and -5.8‰ (PID dataset, Fig 8).

**Fig 9:**
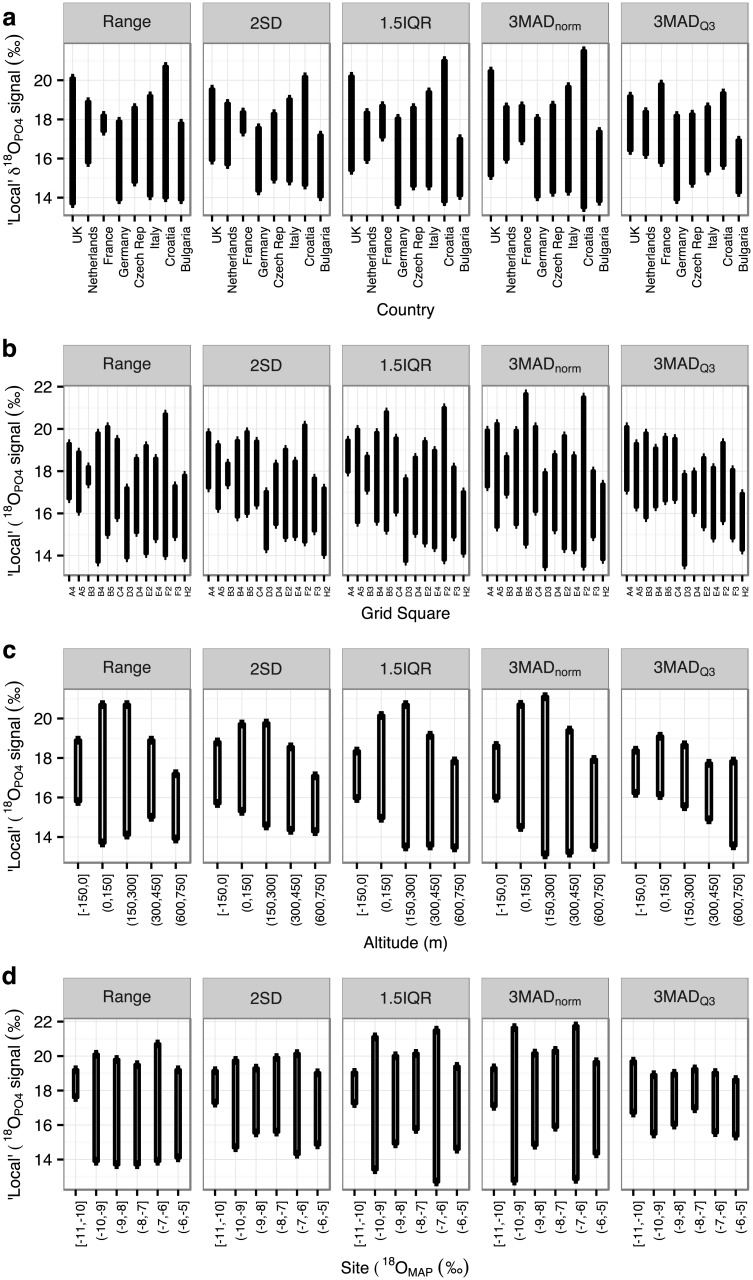
The limits of the ‘local’ signal of the different outlier identification methods for European sites, calculated: (a) by country, (b) by lat/long grid square, (c) by altitude, (d) by site-specific modern precipitation oxygen isotopic values (δ^18^O_MAP_). Plotted for groupings with N>4. Criteria of each method (2SD, 1.5IQR, 3MAD_norm_, 3MAD_Q3_) as defined in the text. (a) Full data in S4 Table, the countries are statistically different: H(8) = 297.67, P<0.001. (b) Full data including alphanumeric codes in S5 Table, the grid squares are statistically different: H(12) = 312.50, P<0.001. (c) Full data in S6, data binned in 150m intervals, the altitudinal groups are statistically different: H(4) = 155.94, P<0.001. (d) Full data in S7 Table, δ^18^O_MAP_ data binned in 1‰ intervals, the precipitation oxygen isotope groups are statistically different: H(3) = 93.78, P<0.001.

However, all of these comparisons include any outliers present. We can instead examine these relationships using the boundaries of the 2SD, 1.5IQR, 3MAD_norm_ and 3MAD_Q3_ methods (i.e. we restrict the comparison to the δ^18^O values that would be defined as ‘local’ by each method). When considering the limits of calculated ‘local’ signal of the different outlier identification methods when the data are grouped by country, latitude/longitude grid square, altitude, and δ^18^O_MAP_, there remains a considerable spread ***and overlap*** of values across most of Europe, whichever way the data are divided (Fig 9).

Given the uniformity that we have highlighted across European PID tooth enamel data, it is noteworthy that in the full European dataset there are a number of individuals with much lower δ^18^O, particularly from two sites, Volders, Austria (range from 5.2 to 16.9‰, N = 129 [72]) and Lake Inari, Finland (range from 11.9 to 13.6‰, N = 4 [49]). As these analyses are of bone phosphate, it would be useful to examine this further using tooth enamel.

## Discussion

### Degree of Intra-Sample Variability

Our results indicate that sample size is an important consideration in experimental design. Notably, sample sizes of less than *c*. 25 individuals give widely varying outlier percentage estimates for all of the statistical methods investigated. We therefore suggest that statistical techniques of outlier labelling should be used with caution for small sample sizes (N<25). Sample size also affects which measures of scale should be chosen for describing the dataset: range is significantly correlated with sample size, whereas MAD_Q3_ is not and the others are correlated only slightly.

The observed values of site range are large and are usually greater than 2‰, in some cases much larger (Fig 1 and S1 Table). When using the more robust methods of describing the data (i.e. 1.5IQR and 3MAD), the spread encompassed within the boundaries is usually greater than 2‰ (Fig 1b and S2 Table). When we consider all sites together, only *c*. 76% of data points fall within 1‰ of their site mean or median (Fig 2). While these data can not and should not be used as a method to determine the amount of variation within a population drinking water from the same source, they do suggest that the typical oxygen isotopic spread for a population would likely be much greater than the 2‰ figure that has been suggested, and that it is potentially very variable between sites (Fig 1, S1 and S2 Tables).

There are several possible explanations for the large variability within human oxygen isotopic values from single archaeological sites. Firstly, these studies were performed upon samples where migrants were expected, which may lead to large variability. Secondly, a methodological problem, such as sampling or pre-treatment, might cause greater isotopic variability. Thirdly, the majority of studies represent historic periods where there is likely to have been relatively large amounts of trade, which could include food and drink. Finally, it could be that humans simply do have a high degree of noise in our biomineral oxygen isotope signals, perhaps due to our physiology or through variations in our diets and food preparation methods. It is likely that more than one of these potential explanations accounts for the variation but without further study of modern human biomineral it is not possible to determine which is the primary factor.

### Comparison of Methods of Identifying Outliers

Our analyses show that the choice of statistical method has a profound impact both on whether outliers are identified at a site, and the proportion that are found.

From our observations on intra-sample variability (Fig 1), we see that most samples have a much wider oxygen isotopic spread than 2‰. Thus, the use of a 2‰ spread is highly likely to make false positive outlier identifications. The migrants identified in past studies where this method has been used should be reassessed.

The 2SD method frequently identifies outliers at a site, but at low percentages. Where sample size is large enough, it tends towards an outlier proportion of 4.6%. We consider that the method may well produce both false positive and false negative identifications depending on the data distribution. With tightly clustered data, it will identify the tails as outliers, irrespective of their actual isotope values. For data with many outliers, the measure of scale (SD) will be distorted by those outliers (since it is a conventional and not a robust measure) and thus produce false negatives. If this method is to be used, one should be very careful in assessing the data distribution and critically evaluating the results.

The 1.5IQR method is conservative in how many outliers are identified, and the IQR itself increases with increasing sample size. The 3MAD_norm_ method is also conservative in the number of outliers identified, and the MAD_norm_ is positively correlated with sample size. The MAD_Q3_ is the only measure of scale not correlated with sample size, however the 3MAD_Q3_ method identifies more outliers that either 1.5IQR or 3MAD_norm_. The proportion of outliers identified is often very high, (e.g. up to 48% at the site of Machu Picchu, Peru (N = 25: [73]), due to the often tiny calculated values of the MAD_Q3_ statistic (Fig 1a) resulting from relatively small sample sizes. MAD_Q3_ should be used in preference to MAD_norm_ where the underlying distribution is not normal [58], however in the case of human δ^18^O values we consider that the underlying population approximates to a normal distribution, as would be expected for biological data (Fig 4), and thus MAD_Q3_ is less appropriate than MAD_norm_.

Based on the above, it is clear that 2‰, 2SD and 3MAD_Q3_ are not appropriate means of identifying outliers from human archaeological oxygen isotope data—not because they identify too many or two few outliers, but because they are based on less statistically valid assumptions. 1.5IQR and 3MAD_norm_ give fairly similar and apparently sensible results. Statistically, the 3MAD_norm_ method is more appropriate because MAD_norm_ is a more robust way of describing the data than the IQR [74], but does the choice between these two methods have a notable impact when applied to human oxygen isotope analysis?

**Fig 10:**
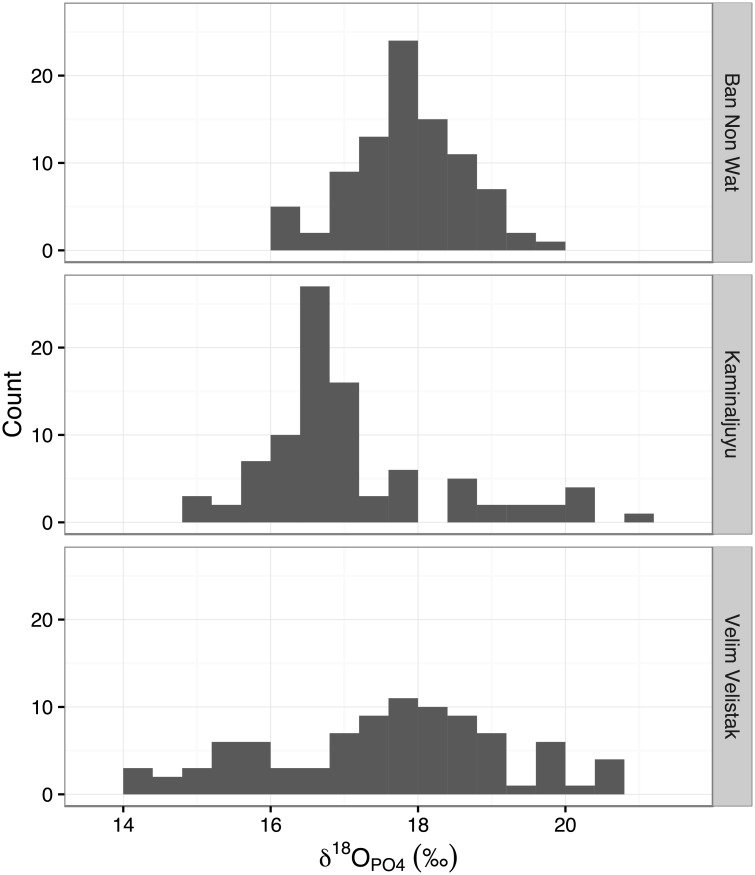
Histograms of data in the Post-Infant Dentition data-subset from the sites of Ban Non Wat, Kaminaljuyu and Velim-Velištak. Data from: Ban Non Wat—King et al. 2013; Kaminaljuyu—White et al. 2000, Wright et al. 1998, Wright et al. 2010; Velim Velištak—Lightfoot et al. 2014. Note that specimens from Kaminaljuyu have been analysed in multiple studies.

We consider the three sites with the highest sample size (*c*. 90) within the PID dataset, Ban Non Wat [75], Kaminaljuyu [38, 76, 77] and Velim Velištak [56] (Table 2, Fig 10) which have very similar mean values but differently shaped distributions (median, range, skewness and kurtosis). For both Ban Non Wat and Velim Velištak no outliers are identified using either 1.5IQR and 3MAD_norm_ methods, whereas at Kaminaljuyu 9 outliers are identified using the 1.5IQR method and 11 using the 3MAD_norm_ method.

**Table 2:** Descriptive statistics for data in the Post-Infant Dentition data-subset from the sites of Velim-Velištak, Kaminaljuyu and Ban Non Wat, in comparison to the numbers of outliers found using the five different outlier identification methods.

Site	N	Mean (‰)	SD (‰)	Median (‰)	Range (‰)	Skewness ±SE	Kurtosis ±SE	Outlier N: 2‰	Outlier N: 2SD	Outlier N: 1.5IQR	Outlier N: 3MAD_norm_	Outlier N: 3MAD_Q3_
Ban Non Wat	89	17.8	0.7	17.8	3.5	-0.16 ±0.26	0.00 ±0.51	17	6	0	0	9
Kaminaljuyu	90	17.0	1.3	16.6	6.2	1.20 ±0.25	1.09 ±0.50	32	7	9	11	22
Velim-Velištak	91	17.5	1.6	17.8	6.6	-0.18 ±0.25	-0.54 ±0.50	50	2	0	0	34

The results using the 1.5IQR and 3MAD_norm_ methods fit well with a visual inspection of the data at all three sites. Velim Velištak has a wide spread of values but none of these are distinct compared to the other data. Ban Non Wat has a smaller spread of values, which approximates a normal distribution, and again none of the values are distinct compared to the other data. On the other hand, Kaminaljuyu has a wide spread of values and the distribution is skewed (mean—median = 0.45‰, skewness = 1.20±0.25). The IQR and MAD_norm_ methods are inefficient with skewed datasets, and specific methods exist for these circumstances (e.g. adjusted boxplots [78] and absolute pairwise differences [79]). However, with human oxygen isotope data, we do not consider skewness to be problematic as the underlying population is highly likely to be normal and skewed data may well indicate a sample with outliers. We can contrast the data from Kaminaljuyu and Velim Velištak: Kaminaljuyu has a smaller range (6.2‰) than Velim Velištak (6.6‰) but the Kaminaljuyu data are skewed whereas the Velim Velištak data are not. From the distribution of the data, it is reasonable to infer that there are outliers at Kaminaljuyu but not at Velim Velištak, and this is what both of the techniques determine.

The two methods produce different estimates of outlier numbers at Kaminaljuyu. The dataset has a tail to the positive side of the distribution but the question is, which of these values deviate significantly enough from the underlying population as to represent outliers? Visual inspection does not make it clear how many outliers there are. The statistical methods tell us that 9 (from 1.5IQR) or 11 (from 3MAD_norm_) outliers are present, and here we come to a subjective judgement as to which estimate represents the real ‘anomalies’–both seem reasonable from the distribution of the data. This is different from whether the numbers of outliers seem reasonable from an archaeological perspective, however, in general, whether the sample has 9 or 11 outliers (out of a sample size of 90) is unlikely to have a meaningful impact on archaeological interpretations.

There are, however, situations where neither of these methods is appropriate. These techniques cannot be used on bimodal data, such as would arise within a population where sub-sets of the population systematically consume different proportions of water with different δ^18^O values. In such a case simple statistical methods applied at the population level are unlikely to be able to produce meaningful estimates of outliers.

In summary, we suggest that the 1.5IQR or 3MAD_norm_ methods are appropriate in most cases—they are robust to outliers and efficient on contaminated data (i.e. data that includes outliers). We acknowledge that they are less efficient on clean normally-distributed data, but they are the most suitable for small and ‘messy’ archaeological datasets. Using these methods is not difficult: the calculations for both methods can be performed using R and SPSS [58] and we would suggest that in archaeological studies both are used and the results compared. In studies combining two isotopic systems, (e.g. oxygen and strontium), the IQR method can be extended to two dimensions by the use of a ‘bagplot’ [80].

### Comparison of Isotope Results between Regions

Our assessment of oxygen isotope studies globally reveals that there is great disparity in the distribution of human oxygen isotope analyses across the world (Fig 5). As with many archaeological distribution maps, this reflects archaeological research, in terms of excavation, bioarchaeologists and publication, rather than the reality of past population distribution. A large number of sites in Europe have been analysed, and a fairly large number of studies from the Americas. Sites in Africa, Asia and Australasia are poorly represented. Even within Europe, the data are biased with the UK being very well-represented with relatively few studies conducted in the rest of Europe (Fig 6). This limits our understanding of the past and highlights the need for more archaeological research in these under-studied regions.

The comparison of the PID data within Europe shows differences between countries, and correlations with latitude, longitude, altitude and δ^18^O_MAP_, as expected given previous studies, but with a high degree of scatter (Fig 8). Thus, when converted to boundaries to delimit outliers, there is a large degree of isotopic overlap regardless of how the data are divided (i.e. by country, latitude and longitude, altitude, δ^18^O_MAP_: Fig 9). Phosphate oxygen isotope values between 16 and 17‰ (or even 14 to 18‰) are very common across Europe, such that an individual with such a value could, theoretically at least, have been born in any of the areas currently studied. There are two potential reasons for this large degree of overlap, and it is likely that both contribute to the problem. Firstly, that human behaviour and physiology lead to large ranges of oxygen isotope values in populations with access to the same drinking water source(s). The typical amount of variation seen in the studies collated here has been discussed above, but is at least 3‰ (S2 Table). Secondly, that the sites studied so far in Europe are in areas with similar precipitation oxygen isotope values (mostly between -9 and -6.5‰). It stands to reason that if the precipitation values are similar then the human values will also be similar. These two problems combined make the application of oxygen isotope studies to Europe difficult, at least in terms of the areas of Europe that have been studied to date: the variation in precipitation mean annual oxygen isotope values across two-thirds of the sites studied so far in Europe is less than the variation one would expect to find in a human population drinking water from the same source. The correlations between human bioapatite δ^18^O and precipitation δ^18^O [17, 18] or drinking (tap) water δ^18^O [16, 49] are apparent over a wide range of δ^18^O_water_, ca. 20‰ [50]. When focussing in on a smaller range, both geographically and in terms of precipitation δ^18^O, the resolving power of the technique is limited as a result of the isotopic spread within a population due to biological noise and the relatively small spread of precipitation δ^18^O across Europe. This has implications for the application of the technique not just in archaeology, but also in forensics and ecology. It would be very interesting to analyse individuals from the south coast of Iberia or northern Scandinavia in order to characterise the full extent of variation that one could expect in human oxygen isotope values within Europe.

Nevertheless, there is some cause for optimism. Weak correlations do exist with latitude, longitude and δ^18^O_MAP_, as has been found in other studies of humans [34]. With a large sample size, small yet significant differences in mean isotope values have been found between groups of individuals from east and west Britain, showing that isotopic differences exist across relatively small geographical areas [46]. Even with the low coverage of data across Europe discussed here, there are areas that have distinct isotope values in the full dataset: Finland, Ireland and Austria have humans with values below 14‰, notably different to the other countries considered (S7 Fig). This would suggest that the technique should work best in areas, such as the Alps and northern Scandinavia, which have distinct precipitation oxygen isotope values compared to the surrounding regions, given a sufficient sample size.

## Conclusion

This study has shown that the range of human δ^18^O values within most archaeological populations is probably greater than 3‰. The reasons for this large variation likely include physiological factors, human modification of drinking water, importation of food and drink, and the use of multiple sources of water with different oxygen isotope values.

When using statistical techniques, both sample size and the choice of statistical method have a profound effect on the number of outliers identified. We recommend the use of both the 1.5IQR and 3MAD_norm_ methods to as large a sample size as is feasible. Care should be taken when using statistical methods where the sample size is less than 25. Researchers should recognise that there is a trade off between sensitivity to outliers and false positive or negative identifications, as well as a degree of subjectivity in the identification of real anomalies (significantly interesting deviations) amongst the noise. All statistical results should be considered in the light of common sense: is the proportion of outliers identified sensible and realistic for the specific archaeological context? All isotopic data are best interpreted in conjunction with all other available evidence, including archaeological and osteological evidence, and complementary isotopic data (e.g. strontium). Parsimonious explanations are to be preferred so as not to present unrealistic scenarios.

Given the large oxygen isotopic variation within archaeological human samples, the limited amount of geographic variation in precipitation oxygen isotope values is challenging. The results presented here show that if one considers a single result from a European individual it is not possible to determine where in Europe that individual is from, as the European regions analysed to date cannot be distinguished solely on the basis of oxygen isotope values. Given this conclusion, any attempt to identify the specific potential homeland of identified migrants is inadvisable. In most circumstances, however, knowing that an individual has migrated a long distance is sufficient to answer the archaeological question posed. When there is other archaeological information to suggest a potential homeland for migrants (see, for example, [55]), stable isotope analysis can refute, but not confirm, such a hypothesis.

Despite these difficulties human oxygen isotope data do, in general, show correlations with geographical parameters. The technique is not fundamentally flawed per se—it is more that the typical variation in human oxygen isotope values is larger than has usually hitherto been assumed.

In summary, statistical techniques can help to identify first-generation migrants from oxygen isotope analysis of an archaeological sample. However, securely identifying migrants is not straightforward. Unless there have been a significant number of previous analyses in an area, each sample should be treated as an individual dataset and in most cases identification of a homeland should not be attempted.

## Supporting Information

S1 Appendix Robustness in measures of scale & Calculation of the median absolute deviation from the median. (PDF)

S1 Fig Comparison of the different measures of scale for each site in the full dataset plotted by site sample size, for all sites where N>4. On the left, (a) the statistic value for the range, SD, IQR and MAD_norm_ and MAD_Q3_. On the right, (b) the spread encompassed by the range, and the boundary values of the methods of 2SD, 1.5IQR, 3MAD_norm_ and 3MAD_Q3_. Criteria of each method (range, 2SD, 1.5IQR, 3MAD_norm_, 3MAD_Q3_) as defined in the text.
(EPS)

S2 Fig Histogram of the difference between each individual’s oxygen isotope value and (a) their site mean oxygen isotopic value or (b) their site median oxygen isotopic value, for all specimens from sites where N>4 in the full dataset. (EPS)

S3 Fig The proportion of outliers found at each site using the five different outlier identification methods, plotted by site sample size, for all sites where N>4 in the full dataset. For some of methods, there are multiple curves apparent for sample sizes between 5 and 25. this is an artefact of producing estimates of either one, two or three outliers within the sample, producing “curves” of 1/x where x is the number of outliers (i.e. 1, 2 or 3).
(EPS)

S4 Fig Density plot of each individual’s oxygen isotopic value expressed as Z score for all specimens from sites where N>4 in the full dataset, with a fitted normal distribution curve overlaid. Z-scores as defined in the text. For the 3820 individuals from 159 sites where N>4, a plot of these z-scores shows that overall, the full dataset z-scores approximate a normal distribution and 174 individuals or 4.56% of the data fall more than 2SD from their site mean.
(EPS)

S5 Fig Map of sites represented in the full dataset. Map created using ArcGIS version 10.2.2 for PC. Source: US National Parks Service.
(TIF)

S6 Fig Map of all European sites represented in the full dataset, with regional modern precipitation oxygen isotopic values indicated. Map created using ArcGIS version 10.2.2 for PC. Precipitation data taken from Bowen 2015 [64] and Bowen and Revenaugh 2003 [65].
(TIF)

S7 Fig Histogram of each individual’s oxygen isotopic value for all European individuals in the full dataset. (EPS)

S8 Fig Oxygen isotope values for all individuals from European sites in the full dataset, plotted by: (a) and (b) country, (c) latitude, (d) longitude, (e) altitude, (f) modern precipitation oxygen isotopic values (δ^18^O_MAP_). (c) Latitude is positively correlated with δ^18^O_PO4_: r_s_ = 0.323, P<0.001, N = 1756. (d) Longitude is negatively correlated with δ^18^O_PO4_: r_s_ = -0.336, P<0.001, N = 1756. (e) Altitude is negatively correlated with δ^18^O_PO4_: r_s_ = -0.337, P<0.001, N = 1756. (f) δ^18^O_MAP_ is positively correlated with δ^18^O_PO4_: r_s_ = 0.190, P<0.001, N = 1756.
(EPS)

S9 Fig The limits of the ‘local’ δ^18^O_PO4_ signal of the different outlier identification methods for European data, calculated: (a) by country, (b) by lat/long grid square, (c) by altitude, (d) by site-specific modern precipitation oxygen isotopic values (δ^18^O_MAP_). Plotted for groupings with N>4. Criteria of each method (range, 2SD, 1.5IQR, 3MAD_norm_, 3MAD_Q3_) as defined in the text. (a) Full data in S4 Table, the countries are statistically different: H(15) = 738.82, P<0.001. (b) Full data including alphanumeric codes in S5 Table, the grid squares are statistically different: H(17) = 631.82, P<0.001. (c) Full data in S6 Table, data binned in 150m intervals, the altitudinal groups are statistically different: H(5) = 545.61, P<0.001. (d) Full data in S7 Table, δ^18^O_MAP_ data binned in 1‰ intervals, the precipitation oxygen isotope groups are statistically different: H(3) = 372.52, P<0.001.
(EPS)

S1 Table Summary of isotopic variation within each site, described using different statistics, with the data grouped by site sample size. (PDF)

S2 Table Isotopic spread encompassed by the boundaries of the different outlier identification methods for each site, with the data grouped by site sample size. (PDF)

S3 Table Descriptive statistics, the isotopic spread of the criteria of the different outlier identification methods, and the numbers and percentage of outliers found using the five different methods for each site in the Post Infant Dentition data subset where N>4. (PDF)

S4 Table The limits of the ‘local’ δ^18^O_PO4_ signal calculated by the different outlier identification methods for each country, for European data. (PDF)

S5 Table The limits of the ‘local’ δ^18^O_PO4_ signal calculated by the different outlier identification methods for each latitude/longitude grid square, for European data. (PDF)

S6 Table The limits of the ‘local’ δ^18^O_PO4_ signal calculated by the different outlier identification methods for European data grouped by altitude. (PDF)

S7 Table The limits of the ‘local’ δ^18^O_PO4_ signal calculated by the different outlier identification methods for European data grouped by site-specific modern precipitation oxygen isotopic values (δ^18^O_MAP_). (PDF)
