# Supplementary material for: On the Use of Biomineral Oxygen Isotope Data to Identify Human Migrants in the Archaeological Record: Intra-Sample Variation, Statistical Methods and Geographical Considerations
Source: PLoS One. 2016 Apr 28;11(4):e0153850. doi: 10.1371/journal.pone.0153850 (PMC4849641; doi:10.1371/journal.pone.0153850)
Supplement: S1 Appendix — (PDF) [file pone.0153850.s001.pdf]

Appendix S1

Robustness in measures of scale

A measure of the robustness of an estimator against outliers is given by the "breakdown point", defined as the smallest percentage of outliers than can cause an estimator to take arbitrary large values (Hampel, 1971; Hampel, 1974). The larger breakdown point an estimator has, the more robust it is. The breakdown point of interquartile range (IQR) is 25%, and that of the median absolute deviation from the median (MAD) is 50%. In contrast, the breakdown point of the standard deviation is 0% - it can be made arbitrarily large by the increase of a single datapoint.
Similarly, estimators of location vary in their degree of robustness. The mean has a breakdown point of 0% (it changes when a single value in the sample set changes), whereas the median has a breakdown point of 50%, the highest possible.

Median absolute deviation from the median

The median absolute deviation from the median is an estimator of the spread in a data, similar to the standard deviation, but is very robust, with approximately a 50% breakdown point, i.e. it is largely unaffected by the presence of extreme values of the data set. It was promoted by Hampel (1974), attributing it to Gauss.

It is given by :
$\text{MAD} = b \text{ median } (|x_i - \text{median}(x)| \; i{=}1,2,...,n)$

The raw MAD is calculated by calculating the absolute difference between each observed value and that of the population median, and then finding the median of these absolute differences. The MAD is then scaled using the constant *b* to make the estimate consistent for the parameter of interest. For a population with a normal distribution, b=1.4826. For data that follows another distribution, then *b* is equal to the inverse of the 75th centile (or Q3) of the raw MAD (Rousseeuw & Croux 1993; Leys et al 2013).

The use of this statistic as an outlier identification method is similar to the SD method, however, the median and MAD are employed instead of the mean and standard deviation. Typically, boundaries defined by the median ±3MAD are used as a cut-off or limit to identify outliers. Since this approach uses two robust estimators (i.e. neither MAD nor the median are unduly affected by extreme values), the interval is seldom inflated, unlike the SD method.

For each site, we calculated two variants of the MAD: one where the MAD was scaled assuming normality of the underlying population (which we term MAD_{norm}), and one where it was scaled using a value of *b* set to 1/Q3 (which we term MAD_{Q3}). In Table S3, for each site we report the raw (unscaled) MAD, the Q3 of that unscaled MAD, the scaled MAD_{norm} and scaled MAD_{Q3}.

The steps to calculate the MAD are as follows (adapted from Leys et al. 2013).
1. Identify the median of the distribution
2. Subtract the median from each observation to create a new series of absolute values (absolute deviations from the median)

3. Rank these absolute values and identify their median (the raw median absolute deviation from the median) and 75th centile (where appropriate).

4a. To produce the MAD_{norm}, scale the raw median absolute deviation from the median by multiplying it by 1.4826.

4b. To produce the MAD_{Q3}, scale the raw median absolute deviation from the median by multiplying it by the inverse of the 75th centile (1/Q3).

References

Hampel FR 1971. A general qualitative definition of robustness, Annals of Mathematical Statistics, 42: 1887–1896.

Hampel, FR 1974: The influence curve and its role in robust estimation. Journal of the American Statistical Association 69: 383-393

Leys C, Ley C, Klein O, Bernard P, and Licata L. 2013. Detecting outliers: Do not use standard deviation around the mean, use absolute deviation around the median. Journal of Experimental Social Psychology 49(4):764-766.

Rousseeuw PJ and Croux C 1993: Alternatives to the median absolute deviation form the median. Journal of the American Statistical Association. 88(424): 1273-1283.
