# Supplementary material for: On the Use of Biomineral Oxygen Isotope Data to Identify Human Migrants in the Archaeological Record: Intra-Sample Variation, Statistical Methods and Geographical Considerations
Source: PLoS One. 2016 Apr 28;11(4):e0153850. doi: 10.1371/journal.pone.0153850 (PMC4849641; doi:10.1371/journal.pone.0153850)
Supplement: S1 Table — (PDF) [file pone.0153850.s011.pdf]

Lightfoot & O'Connell, 2016, Supplementary Tables

Table S1: Summary of isotopic variation within each site, described using different statistics, with the data grouped by site sample size

Site sample size	N	Range				Standard Deviation				Inter-quartile Range				MAD_{norm}				MAD_{Q3}			
		Mean	Med-ian	Min	Max	Mean	Med-ian	Min	Max	Mean	Med-ian	Min	Max	Mean	Med-ian	Min	Max	Mean	Med-ian	Min	Max
**PID data**																					
5-10	34	2.1	1.6	0.5	5.7	0.7	0.5	0.2	1.9	0.8	0.6	0.1	2.4	0.6	0.4	0.1	1.9	0.6	0.6	0.1	1.0
11-20	22	3.1	2.5	1.2	8.1	0.9	0.7	0.4	2.3	1.0	0.9	0.4	2.0	0.8	0.7	0.2	1.5	0.6	0.6	0.3	0.9
21-40	17	2.9	2.5	1.5	7.6	0.8	0.6	0.4	2.0	0.9	0.8	0.4	2.7	0.7	0.5	0.3	2.1	0.6	0.5	0.4	0.8
40+	15	5.1	5.0	3.3	7.1	1.1	1.0	0.7	1.7	1.3	1.2	0.7	2.3	0.9	0.9	0.5	1.5	0.5	0.5	0.4	0.7
**All Data**																					
5-10	64	2.3	1.8	0.4	8.7	0.8	0.7	0.1	3.1	0.8	0.6	0.1	2.1	0.6	0.5	0.0	2.0	0.6	0.7	0.0	1.0
11-20	35	2.9	2.1	1.2	11.6	0.8	0.6	0.4	3.7	1.0	0.7	0.3	4.7	0.7	0.6	0.2	3.9	0.6	0.6	0.3	0.9
21-40	35	4.3	3.3	1.5	10.4	1.0	0.8	0.4	2.4	1.2	0.9	0.4	3.1	0.9	0.7	0.3	2.3	0.6	0.6	0.2	0.8
40+	20	5.2	5.1	2.9	11.9	1.1	1.0	0.6	2.7	1.4	1.2	0.7	4.2	1.0	0.9	0.4	2.9	0.6	0.6	0.4	0.7
