# Supplementary material for: On the Use of Biomineral Oxygen Isotope Data to Identify Human Migrants in the Archaeological Record: Intra-Sample Variation, Statistical Methods and Geographical Considerations
Source: PLoS One. 2016 Apr 28;11(4):e0153850. doi: 10.1371/journal.pone.0153850 (PMC4849641; doi:10.1371/journal.pone.0153850)
Supplement: S2 Table — (PDF) [file pone.0153850.s012.pdf]

Lightfoot & O'Connell, 2016, Supplementary Tables

Table S2: Isotopic spread of the criteria of the different outlier identification methods for each site, with the data grouped by site sample size

Site sample size	N	Range				Standard Deviation				Inter-quartile Range				MAD_{norm}				MAD_{Q3}			
		Mean	Med-ian	Min	Max	Mean	Med-ian	Min	Max	Mean	Med-ian	Min	Max	Mean	Med-ian	Min	Max	Mean	Med-ian	Min	Max
**PID data**																					
5-10	34	2.1	1.6	0.5	5.7	3.0	2.2	0.9	7.5	3.0	2.4	0.4	9.6	3.6	2.7	0.4	11.6	3.6	3.8	0.5	6.0
11-20	22	3.1	2.5	1.2	8.1	3.6	2.8	1.4	9.4	4.0	3.4	1.4	8.0	4.8	4.4	1.3	8.9	3.7	3.7	1.9	5.5
21-40	17	2.9	2.5	1.5	7.6	3.0	2.5	1.4	8.1	3.8	3.2	1.4	10.8	4.2	3.1	1.8	12.5	3.4	3.2	2.3	4.6
40+	15	5.1	5.0	3.3	7.1	4.3	4.2	2.8	6.7	5.1	4.8	2.7	9.2	5.5	5.3	3.1	8.9	3.3	3.3	2.2	4.3
**All Data**																					
5-10	64	2.3	1.8	0.4	8.7	3.2	2.7	0.6	12.3	3.0	2.4	0.4	8.3	3.9	3.1	0.0	12.0	3.5	3.9	0.0	6.0
11-20	35	2.9	2.1	1.2	11.6	3.3	2.4	1.4	14.8	3.8	2.8	1.2	18.8	4.4	3.6	1.3	23.1	3.4	3.5	1.8	5.3
21-40	35	4.3	3.3	1.5	10.4	4.1	3.3	1.4	9.7	4.6	3.6	1.4	12.3	5.1	4.4	1.8	14.0	3.4	3.6	1.4	4.9
40+	20	5.2	5.1	2.9	11.9	4.3	4.0	2.6	10.9	5.4	4.8	2.8	16.7	5.8	5.1	2.7	17.3	3.3	3.4	2.5	4.2
