# Supplementary material for: On the Use of Biomineral Oxygen Isotope Data to Identify Human Migrants in the Archaeological Record: Intra-Sample Variation, Statistical Methods and Geographical Considerations
Source: PLoS One. 2016 Apr 28;11(4):e0153850. doi: 10.1371/journal.pone.0153850 (PMC4849641; doi:10.1371/journal.pone.0153850)
Supplement: S3 Table — (PDF) [file pone.0153850.s013.pdf]

Table S3. Descriptive statistics, the isotopic spread of the criteria of the different outlier identification methods, and the numbers and percentage of outliers found using the five different methods for each site in the Post Infant Dentition data subset where N>4.

Site	N	Mean	SD	Median	Range	Skewness ±SE	Kurtosis ±SE	IQR	MAD un-scaled	Q3 of MAD	MAD_{norm}	MAD_{Q3}	2SD spread (‰)	1.5IQR spread (‰)	$3MAD_{norm}$ spread (‰)	$3MAD_{Q3}$ spread (‰)	Outlier N: 2‰	Outlier N: 2SD	Outlier N: 1.5IQR	Outlier N: $3MAD_{norm}$	Outlier N: $3MAD_{Q3}$	Outlier %: 2‰	Outlier %: 2SD	Outlier %: 1.5IQR	Outlier %: $3MAD_{norm}$	Outlier %: $3MAD_{Q3}$	References
Abingdon	24	18.4	0.4	18.4	1.5	0.12 ±0.47	0.36 ±0.92	0.5	0.22	0.38	0.32	0.57	1.4	1.8	1.9	3.4	0	1	0	0	0	0	4	0	0	0	Chambers, 2006
Anse a la Gourde	6	20.0	0.5	20.0	1.6	0.29 ±0.85	2.41 ±1.74	0.5	0.07	0.76	0.10	0.09	2.0	1.9	0.6	0.6	0	0	0	2	2	0	0	0	33	33	Laffoon et al., 2013
Auldhame	21	17.5	0.6	17.4	2.5	0.46 ±0.50	0.09 ±0.97	1.1	0.50	0.80	0.74	0.63	2.5	4.2	4.4	3.8	1	1	0	0	0	5	5	0	0	0	Evans et al., 2012
Bamburgh	13	16.9	0.7	17.1	2.7	-1.70 ±0.62	3.85 ±1.19	0.7	0.30	0.55	0.44	0.55	2.9	2.8	2.7	3.3	1	1	1	1	1	8	8	8	8	8	Evans et al., 2012
Ban Chiang	39	18.0	1.0	18.2	3.9	-0.48 ±0.38	-0.39 ±0.74	1.5	0.75	1.14	1.11	0.66	4.0	6.0	6.7	3.9	13	2	0	0	2	33	5	0	0	5	Bentley et al., 2005
Ban Lum Khao	10	17.2	1.3	17.2	3.4	-0.21 ±0.69	-1.52 ±1.33	2.4	1.09	1.53	1.62	0.71	5.1	9.7	9.7	4.3	7	0	0	0	0	70	0	0	0	0	Bentley et al., 2009
Ban Non Wat	89	17.8	0.7	17.8	3.5	-0.16 ±0.26	0.00 ±0.51	0.9	0.40	0.91	0.59	0.44	3.0	3.6	3.6	2.7	17	6	0	0	9	19	7	0	0	10	King et al., 2013
Berinsfield	19	17.8	0.4	17.8	1.2	-0.23 ±0.52	-0.85 ±1.01	0.5	0.30	0.40	0.44	0.75	1.4	2.0	2.7	4.5	0	1	0	0	0	0	5	0	0	0	Hughes et al., 2014
Black Gate Newcastle	17	17.4	0.5	17.5	1.7	-0.42 ±0.55	-0.69 ±1.06	0.9	0.40	0.60	0.59	0.67	2.0	3.8	3.6	4.0	1	1	0	0	0	6	6	0	0	0	Evans et al., 2012
Boscombe Down	5	17.4	0.3	17.4	0.7	-0.61 ±0.91	-0.13 ±2.00	0.5	0.21	0.33	0.31	0.65	1.1	2.0	1.9	3.9	0	0	0	0	0	0	0	0	0	0	Evans et al., 2006b
Bowl Hole cemetery	60	18.3	1.0	18.6	4.4	-0.98 ±0.31	0.51 ±0.61	1.3	0.50	0.98	0.74	0.51	3.9	5.0	4.4	3.1	14	3	2	3	8	23	5	3	5	13	Groves et al., 2013
Brownslade	10	18.7	0.4	18.9	1.6	-1.63 ±0.69	4.21 ±1.33	0.3	0.20	0.30	0.30	0.67	1.7	1.3	1.8	4.0	1	1	1	1	0	10	10	10	10	0	Hemer et al., 2013
Caesarea	8	14.7	1.6	14.6	5.7	1.56 ±0.75	3.99 ±1.48	1.1	0.51	1.63	0.75	0.31	6.6	4.4	4.5	1.9	2	1	1	1	2	25	13	13	13	25	Mitchell and Millard, 2009
Cahuachi	11	13.9	2.3	13.0	7.3	1.05 ±0.66	0.23 ±1.28	2.8	0.81	2.20	1.20	0.37	9.4	11.2	7.2	2.2	8	0	0	2	4	73	0	0	18	36	Knudson et al., 2009
Callis Wold	5	17.8	0.8	17.9	2.1	-1.60 ±0.91	3.02 ±2.00	1.3	0.30	1.05	0.44	0.29	3.2	5.0	2.7	1.7	1	0	0	1	1	20	0	0	20	20	Evans et al., 2012
Caspana	11	17.8	0.6	17.9	1.7	0.16 ±0.66	-1.65 ±1.28	1.1	0.57	0.68	0.85	0.84	2.4	4.4	5.1	5.0	0	0	0	0	0	0	0	0	0	0	Knudson and Torres-Rouff, 2009
Catterick	22	17.6	0.5	17.8	1.7	-0.14 ±0.49	-0.89 ±0.95	0.8	0.34	0.67	0.50	0.50	1.9	3.0	3.0	3.0	0	0	0	0	0	0	0	0	0	0	Chenery et al., 2011
Chelsea	22	17.5	0.6	17.4	2.7	0.50 ±0.49	0.85 ±0.95	0.6	0.25	0.55	0.37	0.45	2.6	2.5	2.2	2.7	4	2	3	4	2	18	9	14	18	9	Evans et al., 2012
Clad Hallan	6	18.3	0.3	18.4	0.8	-0.79 ±0.85	-0.14 ±1.74	0.6	0.25	0.33	0.37	0.77	1.2	2.3	2.2	4.6	0	0	0	0	0	0	0	0	0	0	Evans et al., 2012
Cnip	5	17.6	1.0	17.3	2.2	0.63 ±0.91	-1.62 ±2.00	1.9	0.60	1.25	0.89	0.48	3.9	7.4	5.3	2.9	1	0	0	0	1	20	0	0	0	20	Evans et al., 2012
Copan	9	18.1	1.8	18.0	5.0	0.38 ±0.72	-1.10 ±1.40	3.5	1.35	2.34	2.00	0.58	7.2	13.8	12.0	3.5	6	0	0	0	4	67	0	0	0	44	Buikstra et al., 2004
Coventry	10	16.6	0.4	16.6	1.2	0.32 ±0.69	-1.23 ±1.33	0.8	0.35	0.50	0.52	0.70	1.6	3.0	3.1	4.2	0	0	0	0	0	0	0	0	0	0	Evans et al., 2012
Cronk Keeillane	7	17.9	0.6	17.7	1.5	0.72 ±0.79	-0.72 ±1.59	1.2	0.40	0.80	0.59	0.50	2.3	4.8	3.6	3.0	0	0	0	0	0	0	0	0	0	0	Hemer et al., 2014
Dragišić	10	19.2	1.9	19.8	5.5	-1.50 ±0.69	1.27 ±1.33	2.3	0.83	1.57	1.22	0.53	7.5	9.3	7.3	3.2	6	1	0	1	2	60	10	0	10	20	Lightfoot et al., 2014
Duggleby Howe	5	18.0	0.6	17.8	1.4	1.03 ±0.91	0.08 ±2.00	1.0	0.30	0.80	0.44	0.38	2.3	4.0	2.7	2.3	0	0	0	0	0	0	0	0	0	0	Evans et al., 2012
Eastbourne	19	18.0	0.5	18.1	1.7	-0.34 ±0.52	-0.85 ±1.01	0.8	0.40	0.70	0.59	0.57	2.0	3.2	3.6	3.4	0	0	0	0	0	0	0	0	0	0	Evans et al., 2012
El Chorro de Maita	10	18.9	1.1	19.3	3.6	-1.32 ±0.69	1.31 ±1.33	1.5	0.37	1.13	0.54	0.32	4.4	6.0	3.2	1.9	3	1	0	1	2	30	10	0	10	20	Laffoon et al., 2013
Giberville	5	17.9	0.2	18.0	0.5	-0.20 ±0.91	-2.72 ±2.00	0.4	0.20	0.30	0.30	0.67	0.9	1.8	1.8	4.0	0	0	0	0	0	0	0	0	0	0	Brettell et al., 2012
Glauberg	15	16.5	0.6	16.4	2.0	-0.97 ±0.58	0.74 ±1.12	0.7	0.50	0.70	0.74	0.71	2.4	2.8	4.4	4.3	1	1	1	0	0	7	7	7	0	0	Knipper et al., 2014
Glavice	18	16.7	1.1	16.5	3.9	0.34 ±0.54	-0.26 ±1.04	1.5	0.75	1.39	1.10	0.54	4.3	5.8	6.6	3.2	7	1	0	0	3	39	6	0	0	17	Lightfoot et al., 2014
Gloucester	24	18.0	0.7	17.9	2.4	0.16 ±0.47	-1.09 ±0.92	1.3	0.60	0.80	0.89	0.75	2.9	5.0	5.3	4.5	5	0	0	0	0	21	0	0	0	0	Chenery et al., 2010
Hannover-Anderten	5	17.3	0.4	17.0	1.0	0.82 ±0.91	-1.84 ±2.00	0.8	0.10	0.75	0.15	0.13	1.8	3.2	0.9	0.8	0	0	0	2	2	0	0	0	40	40	Brettell et al., 2012
Harappa	12	16.9	0.9	16.8	2.5	0.24 ±0.64	-1.24 ±1.23	1.9	0.75	1.14	1.10	0.65	3.5	7.4	6.6	3.9	5	0	0	0	0	42	0	0	0	0	Kenoyer et al., 2013
Harris Creek	50	20.3	1.0	20.5	5.5	-2.75 ±0.34	9.47 ±0.66	0.9	0.41	0.71	0.60	0.57	4.2	3.5	3.6	3.4	6	2	2	2	2	12	4	4	4	4	Quinn et al., 2008
Hereford	8	17.9	0.5	17.9	1.7	-0.41 ±0.75	1.09 ±1.48	0.7	0.15	0.73	0.22	0.21	2.1	2.6	1.3	1.2	0	0	0	2	3	0	0	0	25	38	Evans et al., 2012
Huaces de Moche	11	16.4	1.7	16.3	5.3	0.39 ±0.66	-0.56 ±1.28	2.5	1.00	2.50	1.48	0.40	6.9	10.0	8.9	2.4	6	0	0	0	5	55	0	0	0	45	Toyne et al., 2014
Hull Magistrates Court	12	15.7	0.8	15.7	3.3	-0.96 ±0.64	2.34 ±1.23	0.9	0.45	0.78	0.67	0.58	3.3	3.6	4.0	3.5	2	1	1	0	1	17	8	8	0	8	Roberts et al., 2013
Isola Sacra	61	16.9	1.0	17.0	5.1	-0.27 ±0.31	0.18 ±0.60	1.4	0.64	1.13	0.95	0.57	4.2	5.4	5.7	3.4	19	4	1	1	8	31	7	2	2	13	Prowse et al., 2007
Kalfata-Budjaka	44	15.6	0.8	15.6	3.9	0.46 ±0.36	0.98 ±0.70	0.8	0.40	0.92	0.59	0.44	3.1	3.1	3.6	2.6	10	3	3	2	4	23	7	7	5	9	Keenleyside et al., 2011

Site	N	Mean	SD	Median	Range	Skewness ±SE	Kurtosis ±SE	IQR	MAD un-scaled	Q3 of MAD	MAD_{norm}	MAD_{Q3}	2SD spread (‰)	1.5IQR spread (‰)	$3MAD_{norm}$ spread (‰)	$3MAD_{Q3}$ spread (‰)	Outlier N: 2‰	Outlier N: 2SD	Outlier N: 1.5IQR	Outlier N: $3MAD_{norm}$	Outlier N: $3MAD_{Q3}$	Outlier %: 2‰	Outlier %: 2SD	Outlier %: 1.5IQR	Outlier %: $3MAD_{norm}$	Outlier %: $3MAD_{Q3}$	References
Kaminaljuyu	90	17.0	1.3	16.6	6.2	1.20 ±0.25	1.09 ±0.50	1.2	0.46	1.16	0.68	0.40	5.2	4.6	4.1	2.4	32	7	9	11	22	36	8	10	12	24	White et al., 2000, Wright and Schwarcz, 1998, Wright et al., 2010
Khok Phanom Di	37	16.6	0.6	16.4	2.9	0.34 ±0.39	0.19 ±0.76	0.8	0.34	0.78	0.50	0.44	2.5	3.0	3.0	2.6	3	2	1	1	1	8	5	3	3	3	Bentley et al., 2007a
Kutna Hora	22	16.4	0.6	16.5	2.4	-0.73 ±0.49	0.36 ±0.95	0.9	0.35	0.68	0.52	0.52	2.5	3.6	3.1	3.1	2	1	0	0	0	9	5	0	0	0	Scheeres et al., 2013
La Tiza	15	15.4	0.8	15.3	2.8	1.40 ±0.58	1.95 ±1.12	0.6	0.32	0.74	0.47	0.43	3.1	2.4	2.8	2.6	2	1	2	2	2	13	7	13	13	13	Buzon et al., 2011, Buzon et al., 2012
Lankhills	46	17.9	1.0	18.1	4.8	-1.33 ±0.35	2.63 ±0.69	0.7	0.35	0.95	0.52	0.37	3.9	3.0	3.1	2.2	10	3	4	4	8	22	7	9	9	17	Eckardt et al., 2009, Evans et al., 2006a
Llandough	15	18.5	0.4	18.5	1.6	-0.39 ±0.58	0.89 ±1.12	0.5	0.20	0.50	0.30	0.40	1.6	2.0	1.8	2.4	0	1	0	1	0	0	7	0	7	0	Hemer et al., 2013
Machu Picchu	25	12.4	2.0	12.0	7.6	0.11 ±0.46	-0.60 ±0.90	2.9	1.35	2.63	2.00	0.51	8.1	11.6	12.0	3.1	17	0	0	0	12	68	0	0	0	48	Turner et al., 2009
Magdalenenberg	45	15.7	0.7	15.7	3.3	-0.21 ±0.35	0.08 ±0.69	1.1	0.50	0.70	0.74	0.71	2.8	4.4	4.4	4.3	6	3	0	0	0	13	7	0	0	0	Oelze et al., 2012
Manzanilla	5	19.2	0.5	19.1	1.3	1.27 ±0.91	2.76 ±2.00	0.7	0.14	0.64	0.21	0.22	1.9	2.8	1.2	1.3	0	0	0	1	1	0	0	0	20	20	Laffoon et al., 2013
Masham	21	16.8	0.5	16.8	1.9	-0.55 ±0.50	0.75 ±0.97	0.6	0.25	0.41	0.37	0.61	1.9	2.4	2.2	3.7	0	2	0	0	0	0	10	0	0	0	Buckberry et al., 2014
Nadin-Gradine	22	18.0	0.8	17.9	3.5	0.83 ±0.49	1.01 ±0.95	1.1	0.59	0.84	0.87	0.69	3.3	4.5	5.2	4.2	5	1	1	0	1	23	5	5	0	5	Lightfoot et al., 2014
Newton plantation	9	17.3	0.8	17.6	2.0	-0.83 ±0.72	-1.21 ±1.40	1.6	0.36	1.23	0.53	0.29	3.2	6.2	3.2	1.8	2	0	0	0	3	22	0	0	0	33	Schroeder et al., 2009
Nin-Ždrijac	48	16.8	1.1	16.8	5.0	-0.19 ±0.34	-0.27 ±0.67	1.7	0.80	1.23	1.19	0.65	4.2	6.6	7.1	3.9	17	2	0	0	2	35	4	0	0	4	Lightfoot et al., 2014
Noen U-loke	21	18.2	0.8	18.2	2.8	0.46 ±0.50	-0.35 ±0.97	0.9	0.39	1.12	0.58	0.35	3.1	3.4	3.5	2.1	6	0	0	0	6	29	0	0	0	29	Cox et al., 2011
Parliament House	6	17.6	0.7	17.4	1.8	1.76 ±0.85	3.29 ±1.74	0.9	0.30	0.63	0.44	0.48	2.6	3.6	2.7	2.9	1	0	0	1	1	17	0	0	17	17	Evans et al., 2012
Peel Castle	11	18.1	0.6	18.0	1.7	0.13 ±0.66	-1.22 ±1.28	1.1	0.50	0.63	0.74	0.79	2.2	4.4	4.4	4.8	0	0	0	0	0	0	0	0	0	0	Hemer et al., 2013, Symonds et al., 2014
Phaeno	14	20.1	0.7	20.2	2.4	0.51 ±0.60	0.48 ±1.15	0.9	0.42	0.75	0.62	0.56	2.6	3.5	3.7	3.3	1	1	0	0	0	7	7	0	0	0	Perry et al., 2009
Pica 8	19	13.1	1.8	13.3	8.1	0.93 ±0.52	3.52 ±1.01	1.4	0.62	1.30	0.92	0.48	7.1	5.6	5.5	2.9	7	1	2	3	4	37	5	11	16	21	Santana-Sagredo et al., 2015
Podvršje-Glavćine	11	17.1	1.1	17.1	3.4	0.15 ±0.66	-1.32 ±1.28	2.0	0.86	1.26	1.28	0.68	4.5	8.0	7.7	4.1	5	0	0	0	0	45	0	0	0	0	Lightfoot et al., 2014
Porthclew	5	18.4	0.5	18.5	1.2	0.16 ±0.91	-1.81 ±2.00	1.0	0.60	0.60	0.89	1.00	2.1	4.0	5.3	6.0	0	0	0	0	0	0	0	0	0	0	Hemer et al., 2013
Radašinovci-Vinogradine	51	18.1	0.9	18.2	4.5	-0.29 ±0.33	-0.05 ±0.66	1.5	0.72	1.09	1.07	0.66	3.7	6.0	6.4	4.0	17	2	0	0	1	33	4	0	0	2	Lightfoot et al., 2014
Radovesice	32	16.3	0.5	16.2	2.4	-0.60 ±0.41	0.74 ±0.81	0.7	0.35	0.60	0.52	0.58	2.1	2.9	3.1	3.5	1	1	1	0	0	3	3	3	0	0	Scheeres et al., 2013
Riccall	12	18.0	0.5	18.3	1.4	-1.10 ±0.64	0.49 ±1.23	0.7	0.15	0.53	0.22	0.29	1.8	2.7	1.3	1.7	1	1	0	2	1	8	8	0	17	8	Evans et al., 2012
Ringlemere	6	18.2	0.4	18.2	1.1	-0.53 ±0.85	1.54 ±1.74	0.5	0.15	0.53	0.22	0.29	1.5	2.0	1.3	1.7	0	0	0	0	0	0	0	0	0	0	Brettell et al., 2012
Rio Muerto	7	12.0	1.8	12.2	5.1	-0.68 ±0.79	-0.05 ±1.59	2.6	1.24	1.86	1.84	0.67	7.0	10.4	11.0	4.0	5	0	0	0	1	71	0	0	0	14	Knudson et al., 2014
SAC	5	14.7	0.3	14.6	0.6	-0.51 ±0.91	-0.61 ±2.00	0.5	0.28	0.31	0.42	0.90	1.0	1.8	2.5	5.4	0	0	0	0	0	0	0	0	0	0	Shaw et al., 2010
Sannerville	6	17.8	0.3	17.8	0.7	-0.26 ±0.85	-0.99 ±1.74	0.6	0.25	0.33	0.37	0.77	1.1	2.2	2.2	4.6	0	0	0	0	0	0	0	0	0	0	Brettell et al., 2012
Schipluiden	7	17.0	1.0	16.7	3.1	1.32 ±0.79	2.49 ±1.59	1.0	0.30	0.90	0.44	0.33	3.9	4.0	2.7	2.0	2	0	0	1	1	29	0	0	14	14	Smits et al., 2010
Šibenik Sv Lovre	25	17.7	1.1	17.5	4.7	1.05 ±0.46	1.91 ±0.90	1.1	0.39	1.02	0.58	0.38	4.4	4.4	3.5	2.3	5	2	2	2	4	20	8	8	8	16	Lightfoot et al., 2014
Singen	5	15.6	0.3	15.7	0.7	-0.44 ±0.91	-2.68 ±2.00	0.6	0.20	0.45	0.30	0.44	1.2	2.4	1.8	2.7	0	0	0	0	0	0	0	0	0	0	Oelze et al., 2012
St Benedict Cemetery	31	17.1	1.0	17.3	3.2	-0.21 ±0.42	-1.09 ±0.82	1.7	0.77	1.14	1.14	0.68	3.9	6.8	6.8	4.1	10	0	0	0	0	32	0	0	0	0	Salesse et al., 2013
St Giles	7	18.2	0.9	18.2	2.7	1.02 ±0.79	1.63 ±1.59	0.9	0.50	1.10	0.74	0.45	3.5	3.6	4.4	2.7	2	0	1	0	1	29	0	14	0	14	Evans et al., 2012
St Johns College	12	16.4	0.8	16.5	3.1	-0.70 ±0.64	1.87 ±1.23	0.8	0.28	0.82	0.41	0.33	3.1	3.2	2.4	2.0	2	1	1	1	2	17	8	8	8	17	Pollard et al., 2012
St Thomas Kirk	8	18.4	0.4	18.3	1.3	1.32 ±0.75	2.17 ±1.48	0.5	0.25	0.38	0.37	0.67	1.6	2.1	2.2	4.0	0	1	0	0	0	0	13	0	0	0	Evans et al., 2012
Swifterbant	8	17.5	0.5	17.3	1.5	2.09 ±0.75	4.73 ±1.48	0.4	0.15	0.35	0.22	0.43	1.9	1.7	1.3	2.6	1	1	1	1	1	13	13	13	13	13	Smits et al., 2010
Teotihuacan	41	17.0	1.7	16.7	7.1	1.00 ±0.37	0.85 ±0.72	1.8	0.90	1.70	1.33	0.53	6.7	7.2	8.0	3.2	19	3	3	2	12	46	7	7	5	29	White et al., 2007, White et al., 2002, White et al., 2004a, White et al., 2004b
Teouma	9	15.3	1.0	15.2	3.4	1.51 ±0.72	3.25 ±1.40	1.1	0.52	0.89	0.77	0.59	4.0	4.4	4.6	3.5	2	1	1	1	1	22	11	11	11	11	Bentley et al., 2007b
Teposcolula	16	12.0	0.7	12.2	2.3	-0.25 ±0.56	-0.82 ±1.09	1.1	0.57	0.83	0.84	0.68	2.7	4.5	5.0	4.1	2	0	0	0	0	13	0	0	0	0	Warriner et al., 2012
Tikal	63	18.9	1.4	19.2	7.0	-0.63 ±0.30	0.34 ±0.59	1.7	0.70	1.74	1.04	0.40	5.8	6.8	6.2	2.4	29	4	1	3	21	46	6	2	5	33	Wright, 2012
Tombos	17	22.8	1.6	22.3	6.3	0.88 ±0.55	1.03 ±1.06	2.2	1.05	1.67	1.56	0.63	6.5	8.6	9.3	3.8	8	1	0	0	2	47	6	0	0	12	Buzon and Bowen, 2010
Tutu	5	19.7	0.5	19.7	1.4	-0.58 ±0.91	0.32 ±2.00	0.9	0.29	0.70	0.43	0.42	2.1	3.8	2.6	2.5	0	0	0	0	0	0	0	0	0	0	Laffoon et al., 2013
Velim-Velištak	91	17.5	1.6	17.8	6.6	-0.18 ±0.25	-0.54 ±0.50	2.4	1.01	2.07	1.50	0.49	6.4	9.6	9.0	2.9	50	2	0	0	34	55	2	0	0	37	Lightfoot et al., 2014

Site	N	Mean	SD	Median	Range	Skewness ±SE	Kurtosis ±SE	IQR	MAD un-scaled	Q3 of MAD	MAD_{norm}	MAD_{Q3}	2SD spread (‰)	1.5IQR spread (‰)	$3MAD_{norm}$ spread (‰)	$3MAD_{Q3}$ spread (‰)	Outlier N: 2‰	Outlier N: 2SD	Outlier N: 1.5IQR	Outlier N: $3MAD_{norm}$	Outlier N: $3MAD_{Q3}$	Outlier %: 2‰	Outlier %: 2SD	Outlier %: 1.5IQR	Outlier %: $3MAD_{norm}$	Outlier %: $3MAD_{Q3}$	References
Vis-Bandirica	5	17.1	1.1	17.3	2.7	-0.78 ±0.91	-0.14 ±2.00	2.0	0.72	1.33	1.07	0.54	4.3	7.8	6.4	3.2	2	0	0	0	1	40	0	0	0	20	Lightfoot et al., 2014
Wadi Halfa	11	25.0	0.6	25.0	1.7	-0.33 ±0.66	-1.33 ±1.28	1.2	0.60	0.70	0.89	0.86	2.6	4.8	5.3	5.1	0	0	0	0	0	0	0	0	0	0	White et al., 2004c
Wasperton	21	18.1	0.5	18.1	1.9	0.44 ±0.50	-0.25 ±0.97	0.8	0.30	0.60	0.44	0.50	1.9	3.0	2.7	3.0	1	1	0	0	0	5	5	0	0	0	Evans et al., 2012
Wetwang/Garton	25	17.4	0.5	17.3	1.8	0.20 ±0.46	-0.42 ±0.90	0.6	0.30	0.55	0.44	0.55	2.0	2.6	2.7	3.3	0	0	0	0	0	0	0	0	0	0	Jay et al., 2013
Weymouth	10	15.5	0.8	15.7	2.9	-1.36 ±0.69	3.21 ±1.33	0.8	0.35	0.68	0.52	0.52	3.1	3.1	3.1	3.1	2	1	1	1	1	20	10	10	10	10	Evans et al., 2012
York	69	17.8	0.9	17.8	5.1	-0.32 ±0.29	1.02 ±0.57	1.3	0.60	0.90	0.89	0.67	3.6	5.0	5.3	4.0	15	3	1	1	1	22	4	1	1	1	Buckberry et al., 2014, Leach et al., 2009, Müldner et al., 2011
Zadar	43	16.6	1.1	16.6	4.6	-0.15 ±0.36	-0.69 ±0.71	1.9	0.94	1.26	1.39	0.75	4.5	7.6	8.4	4.5	19	2	0	0	2	44	5	0	0	5	Lightfoot et al., 2014

References

Bentley, R.A., Buckley, H.R., Spriggs, M., Bedford, S., Ottley, C.J., Nowell, G.M., Macpherson, C.G., Pearson, D.G., 2007b. Lapita migrants in the Pacific's oldest cemetery: Isotopic analysis at Teouma, Vanuatu, American Antiquity 72, 645-656.

Bentley, R.A., Cox, K.J., Tayles, N., Higham, C., Macpherson, C., Nowell, G., Cooper, M., Hayes, T.E.F., 2009. Community Diversity at Ban Lum Khao Thailand: Isotopic Evidence from the Skeletons, Asian Perspectives 48, 79-97.

Bentley, R.A., Pietrusewsky, M., Douglas, M.T., Atkinson, T.C., 2005. Matrilocality during the prehistoric transition to agriculture in Thailand?, Antiquity 79, 865-881.

Bentley, R.A., Tayles, N., Higham, C., Macpherson, C., Atkinson, T.C., 2007a. Shifting gender relations at Khok Phanom Di, Thailand - Isotopic evidence from the skeletons, Current Anthropology 48, 301-314.

Brettell, R., Evans, J., Marzinzik, S., Lamb, A., Montgomery, J., 2012. 'Impious Easterners': Can Oxygen and Strontium Isotopes Serve as Indicators of Provenance in Early Medieval European Cemetery Populations?, European Journal of Archaeology 15, 117-145.

Buckberry, J., Montgomery, J., Towers, J., Müldner, G., Holst, M., Evans, J., Gledhill, A., Neale, N., Lee Thorp, J.A., 2014. Finding Vikings in the Danelaw, Oxford Journal of Archaeology 33, 413-434.

Buikstra, J., Price, T., Burton, J., Wright, L., 2004. Tombs from Copan's Acropolis: A Life History Approach, in: Bell, E., Canuto, M., Sharer, R. (Eds.), Understanding Early Classic Copan, University of Pennsylvania Museum of Archaeology and Anthropology, Philadelphia, pp. 191-212.

Buzon, M.R., Bowen, G.J., 2010. Oxygen And Carbon Isotope Analysis Of Human Tooth Enamel From The New Kingdom Site Of Tombos In Nubia, Archaeometry 52, 855-868.

Buzon, M.R., Conlee, C.A., Bowen, G.J., 2011. Refining Oxygen Isotope Analysis in the Nasca Region of Peru: An Investigation of Water Sources and Archaeological Samples, International Journal of Osteoarchaeology 21, 446-455.

Buzon, M.R., Conlee, C.A., Simonetti, A., Bowen, G.J., 2012. The consequences of Wari contact in the Nasca region during the Middle Horizon: archaeological, skeletal, and isotopic evidence, Journal of Archaeological Science 39, 2627-2636.

Chambers, E.N., 2006. Oxygen Isotope Analysis of Human Tooth Enamel Carbonate: Implications for Climatological and Environmental Research, Department of Archaeology, The Ohio State University, Ohio.

Chenery, C., Eckardt, H., Müldner, G., 2011. Cosmopolitan Catterick? Isotopic evidence for population mobility on Rome's Northern frontier, Journal of Archaeological Science 38, 1525-1536.

Chenery, C., Müldner, G., Evans, J., Eckardt, H., Lewis, M., 2010. Strontium and stable isotope evidence for diet and mobility in Roman Gloucester, UK, Journal of Archaeological Science 37, 150-163.

Cox, K.J., Bentley, R.A., Tayles, N., Buckley, H.R., Macpherson, C.G., Cooper, M.J., 2011. Intrinsic or extrinsic population growth in Iron Age northeast Thailand? The evidence from isotopic analysis, Journal of Archaeological Science 38, 665-671.

Eckardt, H., Chenery, C., Booth, P., Evans, J.A., Lamb, A., Müldner, G., 2009. Oxygen and strontium isotope evidence for mobility in Roman Winchester, Journal of Archaeological Science 36, 2816–2825.

Evans, J.A., Chenery, C.A., Fitzpatrick, A.P., 2006b. Bronze age childhood migration of individuals near Stonehenge, revealed by strontium and oxygen isotope tooth enamel analysis, Archaeometry 48, 309-321.

Evans, J.A., Chenery, C.A., Montgomery, J., 2012. A summary of strontium and oxygen isotope variation in archaeological human tooth enamel excavated from Britain, Journal of Analytical Atomic Spectrometry 27, 754-764.

Evans, J.A., Stoodley, N., Chenery, C., 2006a. A strontium and oxygen isotope assessment of a possible fourth century immigrant population in a Hampshire cemetery, southern England, Journal of Archaeological Science 33, 265-272.

Groves, S.E., Roberts, C.A., Lucy, S., Pearson, G., Grocke, D.R., Nowell, G., Macpherson, C.G., Young, G., 2013. Mobility histories of 7th-9th century AD people buried at Early Medieval Bamburgh, Northumberland, England, American Journal of Physical Anthropology 151, 462-476.

Hemer, K.A., Evans, J.A., Chenery, C.A., Lamb, A.L., 2013. Evidence of early medieval trade and migration between Wales and the Mediterranean Sea region, Journal of Archaeological Science 40, 2352-2359.

Hemer, K.A., Evans, J.A., Chenery, C.A., Lamb, A.L., 2014. No Man is an island: evidence of pre-Viking Age migration to the Isle of Man, Journal of Archaeological Science 52, 242-249.

Hughes, S.S., Millard, A.R., Lucy, S.J., Chenery, C.A., Evans, J.A., Nowell, G., Pearson, D.G., 2014. Anglo-Saxon origins investigated by isotopic analysis of burials from Berinsfield, Oxfordshire, UK, Journal of Archaeological Science 42, 81-92.

Jay, M., Montgomery, J., Nehlich, O., Towers, J., Evans, J., 2013. British Iron Age chariot burials of the Arras culture: a multi-isotope approach to investigating mobility levels and subsistence practices, World Archaeology 45, 473-491.

Keenleyside, A., Schwarcz, H.P., Panayotova, K., 2011. Oxygen isotopic evidence of residence and migration in a Greek colonial population on the Black Sea, Journal of Archaeological Science 38, 2658-2666.

Kenoyer, J.M., Price, T.D., Burton, J.H., 2013. A new approach to tracking connections between the Indus Valley and Mesopotamia: initial results of strontium isotope analyses from Harappa and Ur, Journal of Archaeological Science 40, 2286-2297.

King, C.L., Bentley, R.A., Tayles, N., Viðarsdóttir, U.S., Nowell, G., Macpherson, C.G., 2013. Moving peoples, changing diets: Isotopic differences highlight migration and subsistence changes in the Upper Mun Valley, Thailand, Journal of Archaeological Science 40, 1681-1688.

Knipper, C., Meyer, Jacobi, F., Roth, C., Fecher, M., Stephan, E., Schatz, K., Hansen, L., Posluschny, A., Höppner, B., Maus, M., Pare, C.F.E., Alt, K.W., 2014. Social differentiation and land use at an Early Iron Age "princely seat": bioarchaeological investigations at the Glauberg (Germany), Journal of Archaeological Science 41, 818-835.

Knudson, K.J., Goldstein, P.S., Dahlstedt, A., Somerville, A., Schoeninger, M.J., 2014. Paleomobility in the Tiwanaku diaspora: Biogeochemical analyses at Rio Muerto, Moquegua, Peru, American Journal of Physical Anthropology 155, 405-421.

Knudson, K.J., Torres-Rouff, C., 2009. Investigating Cultural Heterogeneity in San Pedro de Atacama, Northern Chile, Through Biogeochemistry and Bioarchaeology, American Journal of Physical Anthropology 138, 473-485.

Knudson, K.J., Williams, S.R., Osborn, R., Forgey, K., Williams, P.R., 2009. The geographic origins of Nasca trophy heads using strontium, oxygen, and carbon isotope data, Journal of Anthropological Archaeology 28, 244-257.

Laffoon, J.E., Valcarcel Rojas, R., Hofman, C.L., 2013. Oxygen and carbon isotope analysis of human dental enamel from the Caribbean: Implications for investigating individual origins, Archaeometry 55, 742-765.

Leach, S., Lewis, M., Chenery, C., Müldner, G., Eckardt, H., 2009. Migration and Diversity in Roman Britain: A Multidisciplinary Approach to the Identification of Immigrants in Roman York, England, American Journal of Physical Anthropology 140, 546-561.

Lightfoot, E., Slaus, M., O'Connell, T.C., 2014. Water consumption in Iron Age, Roman and Early Medieval Croatia, American Journal of Physical Anthropology 154, 535-543.

Mitchell, P.D., Millard, A.R., 2009. Migration to the Medieval Middle East With the Crusades, American Journal of Physical Anthropology 140, 518-525.

Müldner, G., Chenery, C., Eckardt, H., 2011. The 'Headless Romans': multi-isotope investigations of an unusual burial ground from Roman Britain, Journal of Archaeological Science 38, 280-290.

Oelze, V.M., Koch, J.K., Kupke, K., Nehlich, O., Zauner, S., Wahl, J., Weise, S.M., Rieckhoff, S., Richards, M.P., 2012. Multi-Isotopic Analysis Reveals Individual Mobility and Diet at the Early Iron Age Monumental Tumulus of Magdalenenberg, Germany, American Journal of Physical Anthropology 148.

Perry, M.A., Coleman, D.S., Dettman, D.L., Al-Shiyab, A.H., 2009. An Isotopic Perspective on the Transport of Byzantine Mining Camp Laborers Into Southwestern Jordan, American Journal of Physical Anthropology 140, 429-441.

Pollard, A.M., Ditchfield, P., Piva, E., Wallis, S., Falys, C., Ford, S., 2012. 'Sprouting like cockle amongst the wheat': The St Brice's Day massacre and the isotopic analysis of human bones from St John's College, Oxford, Oxford Journal of Archaeology 31, 83-102.

Prowse, T.L., Schwarcz, H.R., Garnsey, P., Knyf, M., Macchiarelli, R., Bondioli, L., 2007. Isotopic evidence for age-related immigration to imperial Rome, American Journal of Physical Anthropology 132, 510-519.

Quinn, R.L., Tucker, B.D., Krigbaum, J., 2008. Diet and mobility in Middle Archaic Florida: stable isotopic and faunal evidence from the Harris Creek archaeological site (8Vo24), Tick Island, Journal of Archaeological Science 35, 2346-2356.

Roberts, C.A., Millard, A.R., Nowell, G.M., Grocke, D.R., Macpherson, C.G., Pearson, D.G., Evans, D.H., 2013. Isotopic Tracing of the Impact of Mobility on Infectious Disease: The Origin of People With Treponematosis Buried in Hull, England, in the Late Medieval Period, American Journal of Physical Anthropology 150, 273-285.

Salesse, K., Dufour, E., Castex, D., Veleminsky, P., Santos, F., Kucharova, H., Jun, L., Bruzek, J., 2013. Life history of the individuals buried in the St. Benedict cemetery (Prague, 15th-18th centuries): Insights from 14C dating and stable isotope (d13C, d15N, d18O) analysis, American Journal of Physical Anthropology 151, 202-214.

Santana-Sagredo, F., Lee-Thorp, J.A., Schulting, R., Uribe, M., 2015. Isotopic Evidence for Divergent Diets and Mobility Patterns in the Atacama Desert, Northern Chile, During the Late Intermediate Period (AD 900-1450), American Journal of Physical Anthropology 156, 374-387.

Scheeres, M., Knipper, C., Hauschild, M., Schoenfelder, M., Siebel, W., Pare, C., Alt, K.W., 2013. "Celtic migrations": Fact or fiction? Strontium and oxygen isotope analysis of the Czech cemeteries of Radovesice and Kutna Hora in Bohemia, American Journal of Physical Anthropology 155, 496-512.

Schroeder, H., O'Connell, T.C., Evans, J.A., Shuler, K.A., Hedges, R.E.M., 2009. Trans-Atlantic Slavery: Isotopic Evidence for Forced Migration to Barbados, American Journal of Physical Anthropology 139, 547-557.

Shaw, B., Buckley, H., Summerhayes, G., Anson, D., Garling, S., Valentin, F., Mandui, H., Stirling, C., Reid, M., 2010. Migration and mobility at the Late Lapita site of Reber-Rakival (SAC), Watom Island using isotope and trace element analysis: a new insight into Lapita interaction in the Bismarck Archipelago, Journal of Archaeological Science 37, 605-613.

Smits, E., Millard, A.R., Nowell, G., Pearson, D.G., 2010. Isotopic investigation of diet and residential mobility in the Neolithic of the Lower Rhine Basin, European Journal of Archaeology 13, 5-31.

Symonds, L., Price, D., Keenleyside, A., Burton, J., 2014. Medieval Migrations: Isotope Analysis of Early Medieval Skeletons on the Isle of Man, Medieval Archaeology 58, 1-20.

Toyne, J.M., White, C.D., Verano, J.W., Castillo, S.U., Millaire, J.F., Longstaffe, F.J., 2014. Residential histories of elites and sacrificial victims at Huacas de Moche, Peru, as reconstructed from oxygen isotopes, Journal of Archaeological Science 42, 15-28.

Turner, B.L., Kamenov, G.D., Kingston, J.D., Armelagos, G.J., 2009. Insights into immigration and social class at Machu Picchu, Peru based on oxygen, strontium, and lead isotopic analysis, Journal of Archaeological Science 36, 317-332.

Warriner, C., Garcia, N.R., Spores, R., Tuross, N., 2012. Disease, demography, and diet in early colonial new Spain: Investigation of a sixteenth-century Mixtec cemetery at Teposcolula Yucundaa, Latin American Antiquity 23, 467-489.

White, C., Longstaffe, F.J., Law, K.R., 2004c. Exploring the effects of environment, physiology and diet on oxygen isotope ratios in ancient Nubian bones and teeth, Journal of Archaeological Science 31, 233-250.

White, C.D., Price, T.D., Longstaffe, F.J., 2007. Residential Histories Of The Human Sacrifices At The Moon Pyramid, Teotihuacan: Evidence From Oxygen And Strontium Isotopes, Ancient Mesoamerica 18, 159-172.

White, C.D., Spence, M.W., Longstaffe, F.J., Stuart-Williams, H., Law, K.R., 2002. Geographic identities of the sacrificial victims from the Feathered Serpent Pyramid, Teotihuacan: Implications for the nature of state power, Latin American Antiquity 13, 217-236.

White, C.D., Spence, M.W., Longstaffe, F.J., Law, K.R., 2000. Testing the nature of Teotihuacan imperialism at Kaminaljuyu using phosphate oxygen-isotope ratios, Journal of Anthropological Research 56, 535-558.

White, C.D., Spence, M.W., Longstaffe, F.J., Law, K.R., 2004a. Demography and ethnic continuity in the Tlailotlacan enclave of Teotihuacan: the evidence from stable oxygen isotopes, Journal of Anthropological Archaeology 23, 385-403.

White, C.D., Storey, R., Longstaffe, F.J., Spence, M.W., 2004b. Immigration, assimilation, and status in the ancient city of Teotihuacan: Stable isotopic evidence from Tlajinga 33, Latin American Antiquity 15, 176-198.

Wright, L.E., 2012. Immigration to Tikal, Guatemala: Evidence from stable strontium and oxygen isotopes, Journal of Anthropological Archaeology 31, 334-352.

Wright, L.E., Schwarcz, H.P., 1998. Stable Carbon and Oxygen Isotopes in Human Tooth Enamel: Identifying Breastfeeding and Weaning in Prehistory, American Journal of Physical Anthropology 106, 1-18.

Wright, L.E., Valdes, J.A., Burton, J.H., Price, T.D., Schwarcz, H.P., 2010. The children of Kaminaljuyu: Isotopic insight into diet and long distance interaction in Mesoamerica, Journal of Anthropological Archaeology 29, 155-178.
