# Supplementary material for: On the Use of Biomineral Oxygen Isotope Data to Identify Human Migrants in the Archaeological Record: Intra-Sample Variation, Statistical Methods and Geographical Considerations
Source: PLoS One. 2016 Apr 28;11(4):e0153850. doi: 10.1371/journal.pone.0153850 (PMC4849641; doi:10.1371/journal.pone.0153850)
Supplement: S4 Table — (PDF) [file pone.0153850.s014.pdf]

Lightfoot & O'Connell, 2016, Supplementary Tables

Table S4. The limits of the 'local' $\delta^{18}O_{PO4}$ signal calculated by the different outlier identification methods for each country, for European data

Country	N	Min (‰)	Max (‰)	Mean - 2SD (‰)	Mean +2SD (‰)	1.5IQR below Q1 (‰)	1.5IQR above Q3 (‰)	Median - $3MAD_{norm}$ (‰)	Median + $3MAD_{norm}$ (‰)	Median - $3MAD_{Q3}$ (‰)	Median + $3MAD_{Q3}$ (‰)
**PID data**											
Bulgaria	44	13.9	17.8	14.1	17.2	14.1	17.0	13.8	17.4	14.3	16.9
Croatia	324	14.0	20.7	14.6	20.2	13.8	21.0	13.5	21.5	15.7	19.3
Czech Rep	85	14.8	18.6	14.9	18.3	14.6	18.6	14.3	18.7	14.7	18.3
France	11	17.4	18.2	17.4	18.4	17.1	18.7	16.9	18.7	15.8	19.8
Germany	70	13.9	17.9	14.3	17.6	13.7	18.1	14.0	18.1	13.9	18.2
Ireland	1	16.7	16.7								
Italy	61	14.1	19.2	14.9	19.0	14.6	19.4	14.3	19.7	15.4	18.6
Netherlands	15	15.8	18.9	15.7	18.8	16.0	18.4	16.0	18.6	16.2	18.4
UK	655	13.7	20.1	15.9	19.5	15.4	20.2	15.1	20.5	16.4	19.2
**All data**											
Austria	129	5.2	16.9	7.4	16.1	8.2	16.2	7.9	16.7	10.7	13.9
Belgium	9	15.9	17.5	15.8	17.6	15.9	17.5	16.2	17.0	15.9	17.4
Bulgaria	44	13.9	17.8	14.1	17.2	14.1	17.0	13.8	17.4	14.3	16.9
Croatia	329	14.0	20.7	14.6	20.2	13.8	21.0	13.5	21.5	15.7	19.3
Czech Rep	90	14.8	18.6	14.9	18.3	14.6	18.6	14.3	18.7	14.8	18.2
Denmark	5	16.9	18.9	16.4	19.8	15.6	20.8	15.6	21.0	16.1	20.6
Finland	4	11.9	13.6								
France	68	14.5	19.2	15.7	19.0	16.2	18.7	16.1	18.7	15.9	18.9
Germany	128	13.8	19.0	14.4	18.0	14.0	18.5	13.8	18.6	14.5	17.9
Greece	1	16.9	16.9								
Ireland	9	13.1	16.7	12.2	17.1	11.5	17.1	9.5	20.1	12.4	17.2
Italy	61	14.1	19.2	14.9	19.0	14.6	19.4	14.3	19.7	15.4	18.6
Netherlands	34	15.8	18.9	16.2	18.7	16.3	18.6	16.3	18.5	15.7	19.1
Norway	1	15.1	15.1								
Turkey	20	13.1	16.3	13.0	16.0	13.2	15.9	12.2	16.6	12.3	16.5
UK	824	13.7	20.3	15.9	19.5	15.6	20.0	15.1	20.5	16.3	19.3
