# Supplementary material for: On the Use of Biomineral Oxygen Isotope Data to Identify Human Migrants in the Archaeological Record: Intra-Sample Variation, Statistical Methods and Geographical Considerations
Source: PLoS One. 2016 Apr 28;11(4):e0153850. doi: 10.1371/journal.pone.0153850 (PMC4849641; doi:10.1371/journal.pone.0153850)
Supplement: S5 Table — (PDF) [file pone.0153850.s015.pdf]

Table S5a. The limits of the 'local' $\delta^{18}O_{PO4}$ signal calculated by the different outlier identification methods for each latitude/longitude grid square, for European data from the PID data sub-set

Latitude	Longitude	Grid Sq	N	Min (‰)	Max (‰)	Mean -2SD (‰)	Mean +2SD (‰)	1.5IQR below Q1 (‰)	1.5IQR above Q3 (‰)	Median - $3MAD_{norm}$ (‰)	Median + $3MAD_{norm}$ (‰)	Median - $3MAD_{Q3}$ (‰)	Median + $3MAD_{Q3}$ (‰)
**PID data**													
50-55	-10- -5	A4	14	16.7	19.3	17.2	19.8	18.0	19.5	17.3	19.9	17.1	20.1
55-60	-10- -5	A5	20	16.1	18.9	16.2	19.3	15.6	20.0	15.4	20.2	16.3	19.3
45-50	-5-0	B3	11	17.4	18.2	17.4	18.4	17.1	18.7	16.9	18.7	15.8	19.8
50-55	-5-0	B4	483	13.7	19.8	15.8	19.4	15.6	19.8	15.5	19.9	16.3	19.1
55-60	-5-0	B5	106	15.0	20.1	16.0	19.9	15.2	20.8	14.5	21.7	16.6	19.6
50-55	0-5	C4	40	15.8	19.5	16.4	19.4	16.1	19.6	16.1	20.1	16.7	19.5
45-50	5-10	D3	50	13.9	17.2	14.3	17.0	13.7	17.6	13.5	17.9	13.6	17.8
50-55	5-10	D4	28	15.1	18.6	15.5	18.3	15.1	18.7	15.2	18.8	16.0	18.0
40-45	10-15	E2	61	14.1	19.2	14.9	19.0	14.6	19.4	14.3	19.7	15.4	18.6
50-55	10-15	E4	63	14.8	18.6	14.9	18.5	14.4	19.0	14.3	18.7	14.8	18.2
40-45	15-20	F2	324	14.0	20.7	14.6	20.2	13.8	21.0	13.5	21.5	15.7	19.3
45-50	15-20	F3	22	14.9	17.3	15.2	17.7	14.9	18.2	14.9	18.0	14.8	18.1
40-45	25-30	H2	44	13.9	17.8	14.1	17.2	14.1	17.0	13.8	17.4	14.3	16.9

Lightfoot & O'Connell, 2016, Supplementary Tables

Table S5b. The limits of the 'local' $\delta^{18}O_{PO4}$ signal calculated by the different outlier identification methods for each latitude/longitude grid square, for European data from the full dataset

Latitude	Longitude	Grid Sq	N	Min (‰)	Max (‰)	Mean -2SD (‰)	Mean +2SD (‰)	1.5IQR below Q1 (‰)	1.5IQR above Q3 (‰)	Median - $3MAD_{norm}$ (‰)	Median + $3MAD_{norm}$ (‰)	Median - $3MAD_{Q3}$ (‰)	Median + $3MAD_{Q3}$ (‰)
**All Data**													
50-55	-10--5	A4	22	13.1	19.3	12.7	21.3	9.2	24.5	15.4	21.2	17.5	19.1
55-60	-10--5	A5	34	16.1	19.1	16.4	19.2	15.8	20.0	15.4	20.2	17.0	18.6
40-45	-5-0	B2	1	18.3	18.3								
45-50	-5-0	B3	11	17.4	18.2	17.4	18.4	17.1	18.7	16.9	18.7	15.8	19.8
50-55	-5-0	B4	606	13.7	19.8	15.9	19.4	15.7	19.7	15.5	19.9	16.4	19.0
55-60	-5-0	B5	137	15.0	20.3	16.0	19.9	15.2	20.8	14.9	21.1	16.5	19.5
45-50	0-5	C3	1	17.3	17.3								
50-55	0-5	C4	50	15.8	19.5	16.0	19.4	15.3	20.0	15.3	20.6	16.5	19.5
45-50	5-10	D3	162	13.8	19.2	14.4	18.5	13.5	19.4	12.8	20.0	14.7	18.1
50-55	5-10	D4	47	15.1	18.6	15.8	18.6	15.9	18.7	16.0	18.6	16.1	18.6
40-45	10-15	E2	61	14.1	19.2	14.9	19.0	14.6	19.4	14.3	19.7	15.4	18.6
45-50	10-15	E3	130	5.2	16.9	7.4	16.2	8.2	16.2	7.9	16.8	10.8	14.0
50-55	10-15	E4	65	14.8	18.6	14.9	18.5	14.5	18.9	14.3	18.7	14.8	18.2
55-60	10-15	E5	6	15.1	18.9	14.7	20.5	14.6	21.1	13.7	22.1	15.7	20.1
40-45	15-20	F2	329	14.0	20.7	14.6	20.2	13.8	21.0	13.5	21.5	15.7	19.3
45-50	15-20	F3	25	14.9	17.3	15.2	17.7	15.0	18.2	14.7	18.3	14.5	18.5
35-40	20-25	G1	1	16.9	16.9								
40-45	25-30	H2	44	13.9	17.8	14.1	17.2	14.1	17.0	13.8	17.4	14.3	16.9
65-70	25-30	H7	4	11.9	13.6								
35-40	35-40	J1	20	13.1	16.3	13.0	16.0	13.2	15.9	12.2	16.6	12.3	16.5
