# Supplementary material for: On the Use of Biomineral Oxygen Isotope Data to Identify Human Migrants in the Archaeological Record: Intra-Sample Variation, Statistical Methods and Geographical Considerations
Source: PLoS One. 2016 Apr 28;11(4):e0153850. doi: 10.1371/journal.pone.0153850 (PMC4849641; doi:10.1371/journal.pone.0153850)
Supplement: S6 Table — (PDF) [file pone.0153850.s016.pdf]

Lightfoot & O'Connell, 2016, Supplementary Tables

Table S6. The limits of the 'local' $\delta^{18}O_{PO4}$ signal calculated by the different outlier identification methods for European data grouped by altitude

Altitude group (m asl)	N	Min (‰)	Max (‰)	Mean - 2SD (‰)	Mean +2SD (‰)	1.5IQR below Q1 (‰)	1.5IQR above Q3 (‰)	Median - $3MAD_{norm}$ (‰)	Median + $3MAD_{norm}$ (‰)	Median - $3MAD_{Q3}$ (‰)	Median + $3MAD_{Q3}$ (‰)
**PID data**											
≤ 10	15	15.8	18.9	15.7	18.8	16.0	18.4	16.0	18.6	16.2	18.4
11 - 150	958	13.7	20.7	15.3	19.7	15.0	20.2	14.5	20.7	16.1	19.1
151 - 300	225	14.1	20.7	14.6	19.8	13.5	20.7	13.1	21.1	15.5	18.7
301 - 450	23	15.0	18.9	14.3	18.6	13.6	19.2	13.2	19.4	14.9	17.7
601+	45	13.9	17.2	14.3	17.1	13.5	17.9	13.5	17.9	13.6	17.8
**All data**											
≤ 10	18	15.8	18.9	15.8	18.9	16.1	18.7	15.7	18.9	16.0	18.6
11 - 150	1175	11.9	20.7	15.2	19.8	15.0	20.2	14.5	20.7	16.1	19.1
151 - 300	278	14.1	20.7	14.8	19.6	14.0	20.3	13.6	20.8	15.7	18.7
301 - 450	50	13.8	18.9	14.6	18.7	13.5	19.7	14.2	19.6	15.7	18.1
451 - 600	156	5.2	18.1	7.5	17.2	8.1	17.0	7.7	17.5	10.9	14.3
601+	79	13.9	19.0	14.2	17.6	13.8	17.8	14.1	17.7	14.5	17.3
