# Supplementary material for: On the Use of Biomineral Oxygen Isotope Data to Identify Human Migrants in the Archaeological Record: Intra-Sample Variation, Statistical Methods and Geographical Considerations
Source: PLoS One. 2016 Apr 28;11(4):e0153850. doi: 10.1371/journal.pone.0153850 (PMC4849641; doi:10.1371/journal.pone.0153850)
Supplement: S7 Table — (PDF) [file pone.0153850.s017.pdf]

Table S7: The limits of the 'local' $\delta^{18}O_{PO4}$ signal calculated by the different outlier identification methods for European data grouped by site-specific modern precipitation oxygen isotopic values ($\delta^{18}O_{MAP}$).

$\delta^{18}O_{MAP}$ group	N	Min (‰)	Max (‰)	Mean - 2SD (‰)	Mean +2SD (‰)	1.5IQR below Q1 (‰)	1.5IQR above Q3 (‰)	Median - $3MAD_{norm}$ (‰)	Median + $3MAD_{norm}$ (‰)	Median - $3MAD_{Q3}$ (‰)	Median + $3MAD_{Q3}$ (‰)
**PID data**											
-11 to -10	10	17.6	19.2	17.3	19.1	17.3	19.1	17.1	19.3	16.7	19.7
-10 to -9	184	13.9	20.1	14.7	19.7	13.4	21.1	12.8	21.6	15.5	18.9
-9 to -8	510	13.7	19.8	15.5	19.3	14.9	20.0	14.8	20.2	16.0	19.0
-8 to -7	151	13.7	19.5	15.6	19.9	15.8	20.2	15.9	20.3	16.9	19.3
-7 to -6	350	13.9	20.7	14.3	20.1	12.7	21.5	12.9	21.7	15.6	19.0
-6 to -5	61	14.1	19.2	14.9	19.0	14.6	19.4	14.3	19.7	15.4	18.6
**All data**											
-14 to -13	4	11.9	13.6								
-11 to -10	12	15.1	19.2	16.0	19.9	17.1	19.0	17.0	19.2	17.4	18.9
-10 to -9	405	5.2	20.3	9.5	21.5	7.0	24.2	7.8	24.8	14.7	17.9
-9 to -8	731	13.1	19.8	15.4	19.3	15.0	19.8	14.7	20.1	15.9	18.9
-8 to -7	186	13.1	19.5	14.5	20.3	14.0	21.0	14.8	21.0	16.7	19.1
-7 to -6	357	13.9	20.7	14.3	20.1	12.7	21.5	12.9	21.7	15.5	19.1
-6 to -5	61	14.1	19.2	14.9	19.0	14.6	19.4	14.3	19.7	15.4	18.6
